# Conflicting Evolutionary Patterns Due to Mitochondrial Introgression and Multilocus Phylogeography of the Patagonian Freshwater Crab *Aegla neuquensis*

**DOI:** 10.1371/journal.pone.0037105

**Published:** 2012-06-07

**Authors:** Brian R. Barber, Jiawu Xu, Marcos Pérez-Losada, Carlos G. Jara, Keith A. Crandall

**Affiliations:** 1 Department of Biology, Brigham Young University, Provo, Utah, United States of America; 2 Instituto de Zoología, Universidad Austral de Chile, Casilla, Valdivia, Chile; 3 Monte L. Bean Life Science Museum, Brigham Young University, Provo, Utah, United States of America; 4 CIBIO, Centro de Investigação em Biodiversidade e Recursos Genéticos, Universidade do Porto, Campus Agrário de Vairão, Vairão, Portugal; Biodiversity Insitute of Ontario - University of Guelph, Canada

## Abstract

**Background:**

Multiple loci and population genetic methods were employed to study the phylogeographic history of the Patagonian freshwater crab *Aegla neuquensis* (Aeglidae: Decopoda). This taxon occurs in two large river systems in the Patagonian Steppe, from the foothills of the Andes Mountains east to the Atlantic Ocean.

**Methodology/Principal Findings:**

A nuclear phylogeny and multilocus nested clade phylogeographic analysis detected a fragmentation event between the Negro and Chico-Chubut river systems. This event occurred approximately 137 thousand years ago. An isolation-with-migration analysis and maximum-likelihood estimates of gene flow showed asymmetrical exchange of genetic material between these two river systems exclusively in their headwaters. We used information theory to determine the best-fit demographic history between these two river systems under an isolation-with-migration model. The best-fit model suggests that the Negro and the ancestral populations have the same effective population sizes; whereas the Chico-Chubut population is smaller and shows that gene flow from the Chico-Chubut into the Negro is four times higher than in the reverse direction. Much of the Chico-Chubut system appears to have only been recently colonized while the Negro populations appear to have been in place for most of the evolutionary history of this taxon.

**Conclusions/Significance:**

Due to mitochondrial introgression, three nuclear loci provided different phylogeographic resolution than the three mitochondrial genes for an ancient fragmentation event observed in the nuclear phylogeny. However, the mitochondrial locus provided greater resolution on more recent evolutionary events. Our study, therefore, demonstrates the need to include both nuclear and mitochondrial loci for a more complete understanding of evolutionary histories and associated phylogeographic events. Our results suggest that gene flow between these systems, before and after fragmentation was through periodic paleolakes that formed in the headwaters region. Fragmentation between the Negro and Chico-Chubut systems was driven by the disappearance of these paleolakes during the Patagonian Glaciation.

## Introduction

Phylogeographic inference is a powerful tool for understanding an organism’s evolutionary history [1], [2]. Mitochondrial markers have been the primary data for most of the discipline’s history [3]. Mitochondrial loci dominance in the field of phylogeography has been recently challenged because this marker provides only a portion of and possibly misleading picture of the history of the organism under study [4]. Despite these concerns mitochondrial markers are and will continue to be the data of choice for discovering phylogeographic patterns [5]. However, independent markers, mostly in the form of nuclear loci are required to confirm mitochondrial patterns and provide more robust estimates of processes, such as gene flow and demographic changes [6], [7]. In this study we employ both mitochondrial and nuclear markers to infer the evolutionary history of a geographically widespread Patagonian organism, the freshwater crab *Aegla neuqeunsis* (Aeglidae: Decopoda).

**Figure 1:**
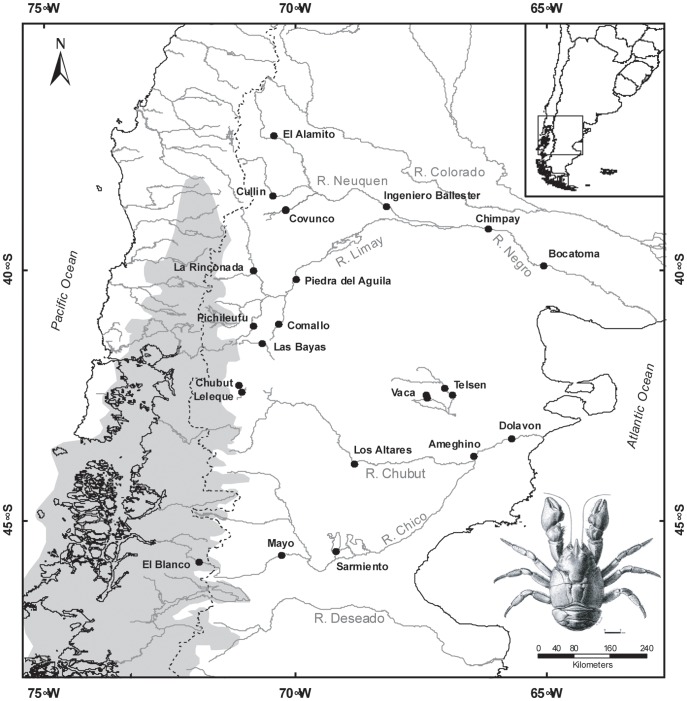
Map showing the sampling locations for *Aegla neuquensis*. The gray area indicates the ice sheet during the last glacial maximum (LGM: 23–24 ka). A picture of *A. neuquensis* is shown.

*Aegla neuquensis* is found in two major river systems (Negro and Chico-Chubut) in the Argentinian Steppe (Fig1: [8]). The Negro system is comprised of three rivers, Neuquen, Limay and Negro where as the Chico and Chubut rivers form the Chico-Chubut system. These two river systems are geographically distant from one another along most of their lengths (∼500 km). Only in the headwater regions of the Limay and Chubut rivers, in the foothills of the Andes, do these two systems come into close proximity (∼2 km) to one another. The headwater region is the only part of the distribution of *Aegla neuquensis* that was directly impacted by glacial ice during glacial cycles. A paleolake called Lake Cari Laufquen may have formed in the region (∼41°S) during the last several glacial cycles (i.e., ∼23–25 ka and 0.7 Ma) [9]. Lake Cari Laufquen would have provided suitable habitat for gene flow between systems [9]. Gene flow between these two systems has been proposed in several fish groups [10]–[14] as well as other decapods [15]. Beyond the limits of glacial ice changes in stream dynamics could have affected the evolutionary history of this taxon. For example, gene flow could also have occurred via the deltoic mosaic that occurred on the continental shelf exposed during previous glacial periods [16], [17].

Five major glacial events occurred in the region over the last four million years [18], [19], [20], [21]: A widespread glaciation of the middle-Pliocene (∼3.5 Ma); The largest Patagonian glacial advance (1.1 Ma); The coldest Pleistocene glaciation (∼0.7 Ma); The last southern Patagonian glaciation that reached the Atlantic coast (180 ka); and lastly the last glacial maximum (LGM), which occurred between 23–25 ka years ago. Each of these periods may have played a role in shaping the phylogeographic history of *Aegla neuquensis* in general and formed paleolakes near the headwaters specifically. However we predict little impact of glaciation on this taxon because most of the distribution of *Aegla neuquensis* occurs beyond the limits of glacial ice. In these ice-free regions, we expect to recover stable demographic histories and genetic structuring between rivers and populations. In contrast we predict glacial cycles will have significantly impacted the headwaters region of this taxon. In this region we expect to recover the genetic signature of historical fragmentation when no paleolakes were present and when there were paleolakes in the region gene flow between the Negro and Chico-Chubut systems. Because the signature of previous glacial cycles is likely to be overwritten by later events, we expect these events to date to recent cycles [20], [22].

The natural history of *Aegla* crabs makes them excellent candidates for phylogeographyic study. *Aegla* species in general occur in freshwater habitats throughout the Patagonian region [21]–[27]. These crabs are small (<60 mm) and spawn from February to March. Females are fecund, laying as many as 1500 eggs. Once laid, eggs attach to the females pleopod where they remain until they hatch approximately six to eight months later [24]. Even after hatching, young crabs remain with the female for several days. The absence of a free-floating larval stage and the long-period of attachment to the female suggests that dispersal is restricted [24]. Limited dispersal and widespread distributions make *Aegla*, including *A. neuquensis* ideal subjects of phylogeographic study [24] and good indicators on how abiotic events have shaped the freshwater systems where it occurs.

We employ three nuclear and one mitochondrial (three genes) locus and a variety of population genetic approaches to recover phylogeographic patterns and processes in *Aegla neuquensis*. Comparing patterns and process across multiple markers will allow us to make rigorous inferences [6], [28], [29] and will provide the most detailed examination of the evolutionary history of a Patagonian freshwater taxon to date and add to our growing understanding of the evolutionary history of the region [16], [17], [30]–[33].

## Results

### Sampling and Sequence Data

**Figure 2:**
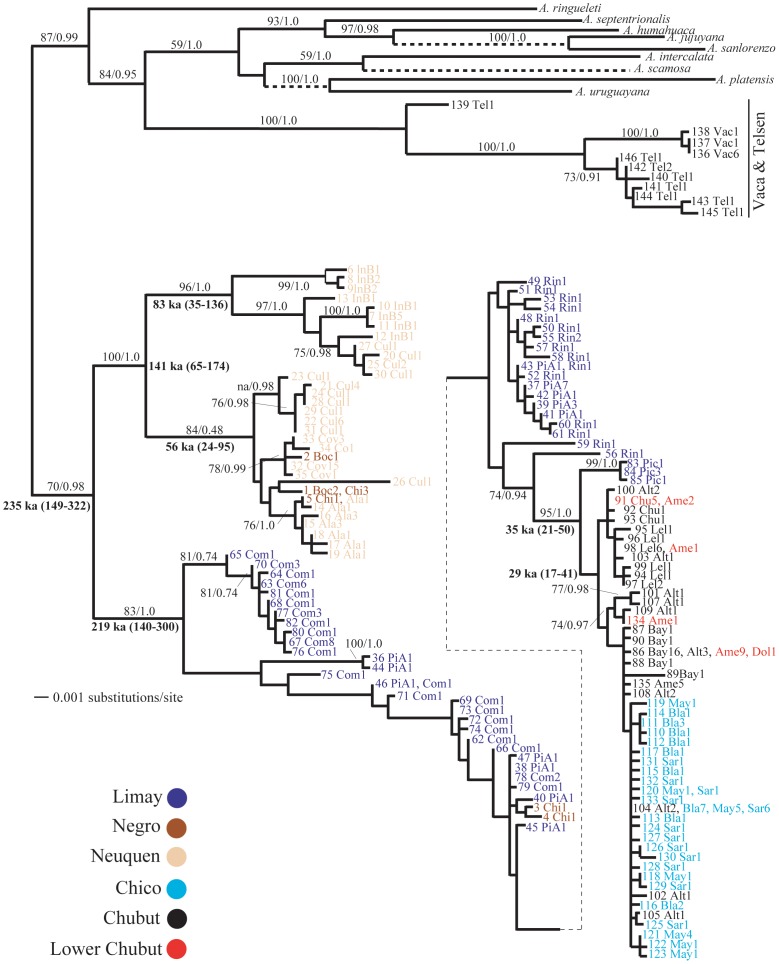
ML trees based on mtDNA haplotypes *Aegla neuquensis*. Numbers above branches are ML bootstrap supports (pre/) and Bayesian posterior probabilities (post/). Numbers in bold are estimates of the time of most recent common ancestor TMRCA [ka (95% HPD intervals)]. Dashed lines indicate outgroups with branches that were arbitrarily shorted to fit on page. Locality (see Table 1) and number of individuals are shown after that haplotype.

**Figure 3:**
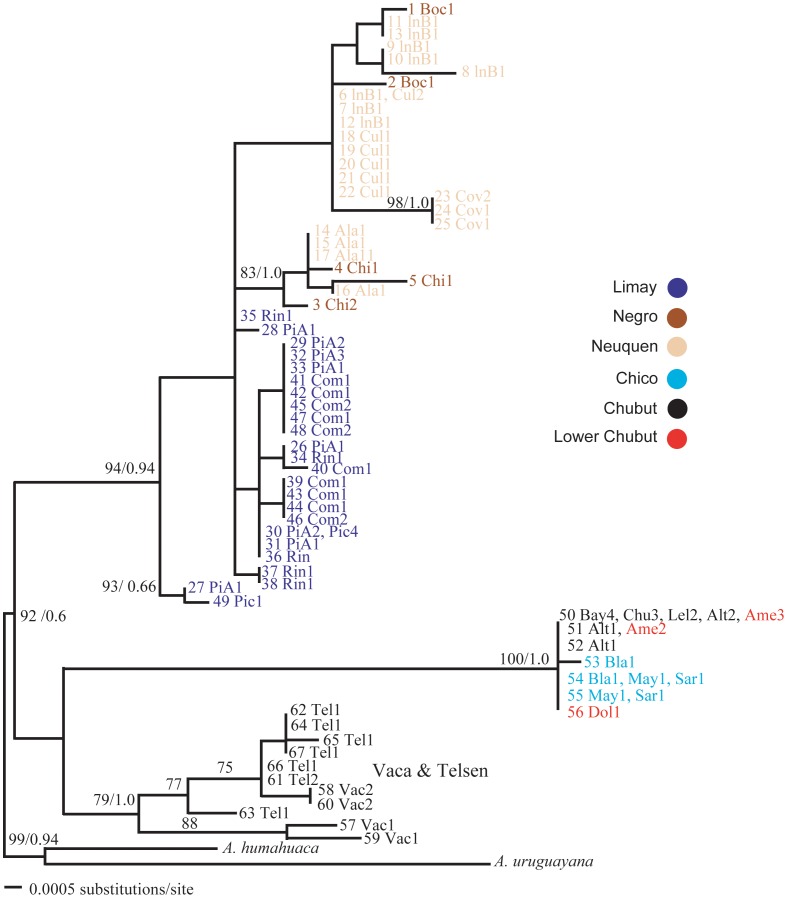
ML trees based on concatenated nDNA haplotypes of *Aegla neuquensis*. Numbers above branches are ML bootstrap supports (pre/) and Bayesian posterior probabilities (post/). Locality (see Table 1) and number of individuals are shown after that haplotype. Color codes indicate the six major rivers.

Mitochondrial and nuclear haplotype diversity was high. We recovered 146 unique haplotypes from 295 individuals in our combined mtDNA data set (Table 1and Fig. 2). Most of the haplotypes (109 of 146, ∼75%) were singletons and only nine occurred in more than one location. The complete mtDNA alignment of fragments of the 16 S, COI, and COII genes is 1699 bp long. The COI (659-bp), COII (568-bp) and 16 S (472-bp) fragments delineated 82, 79 and 39 haplotypes, with 97, 91 and 41 variable sites, respectively, with an average sequence divergence across genes of 2.7% (0–8.5%). In a 1395-bp concatenated alignment of nDNA fragments, 67 haplotypes were identified from 103 crabs (Fig. 3), with an average sequence divergence across loci of 0.6% (0–1.4%). As in the mtDNA, most of the haplotypes (51 of 67, ∼76%) were singletons and only six occur in more than one location. The length of EF1α exon, EF1α intron and ANT intron were 640, 370 and 385 bp respectively defining 13, 21 and 22 haplotypes (phased alleles).

**Table 1:** Sampling locality, sample size (*N*), unique haplotype number (*Hn*), haplotype diversity (*Hd*) and current (θ_π_) and historical (θ_w_) genetic diversity of *Aegla neuquensis* across all mitochondrial genes.

River	Locality	Abbr.	Lat. (S)	Long. (W)	*N*	*Hn*	*Hd*	θ_π_	θ_w_
Aysén	El Blanco	Bla	45°48′24′′	71°54′57′′	18	9	0.837	0.0008	0.0012
Chico	Mayo	May	45°40′56′′	70°15′55′′	14	7	0.824	0.0008	0.0011
Chico	Sarmiento	Sar	45°36′54′′	69°10′57′′	17	12	0.89	0.001	0.0021
Chubut	Los Altares	Alt	43°51′17′′	68°49′04′′	16	11	0.95	0.0026	0.0032
Chubut	Ameghino	Ame	43°41′23′′	66°26′39′′	18	5	0.693	0.0012	0.0014
Chubut	Las Bayas	Bay	41°27′40′′	70°39′25′′	20	5	0.368	0.0005	0.0013
Chubut	Chubut	Chu	42°17′08′′	71°07′15′′	7	3	0.524	0.0003	0.0005
Chubut	Dolavon	Dol	43°20′56′′	65°40′43′′	1	1	–	–	–
Chubut	Leleque	Lel	42°24′41′′	71°04′25′′	12	6	0.758	0.0008	0.0012
Limay	Comallo	Com	41°04′11′′	70°20′19′′	39	22	0.933	0.008	0.0069
Limay	Piedra del Aguila	PiA	40°10′51′′	69°58′47′′	20	12	0.874	0.0094	0.0105
Limay	Pichileufu	Pic	41°06′23′′	70°50′23′′	5	3	0.7	0.0005	0.0006
Limay	La Rinconada	Rin	39°59′49′′	70°50′08′′	16	15	0.992	0.0045	0.0068
Negro	Bocatoma	Boc	39°53′43′′	65°02′51′′	3	2	0.667	0.0032	0.0032
Negro	Chimpay	Chi	39°09′49′′	66°08′29′′	6	4	0.8	0.0158	0.0135
Neuquen	El Alamito	Ala	37°15′20′′	70°25′14′′	11	7	0.891	0.0017	0.002
Neuquen	Covunco	Cov	38°47′39′′	70°11′46′′	20	4	0.432	0.0004	0.0007
Neuquen	Cullin	Cul	38°30′35′′	70°27′11′′	21	12	0.829	0.0092	0.0087
Neuquen	Ingeniero Ballester	InB	38°43′40′′	68°10′23′′	14	8	0.868	0.0099	0.0076
Telsen	Telsen	Tel	42°20′58′′	67°01′31′′	9	8	0.972	0.0066	0.0089
Vaca	Vaca	Vac	42°30′00′′	67°21′35′′	8	3	0.464	0.0004	0.0007

### Phylogenetic Relationships

Unexpectedly, the phylogeographic resolution of mitochondrial and concatenated nuclear phylogenies differed significantly (Figs. 2, 3). The mitochondrial phylogeny recovered a non-monophyletic Limay river with Chico-Chubut haplotypes embedded within it. In contrast, the nuclear phylogeny recovered a monophyletic Chico-Chubut system suggesting that this system has been genetically isolated from the Negro system.

Similar mtDNA phylogenies were recovered using Maximum likelihood and Bayesian methods (Fig. 2). A McDonald-Kreitman test for selection was not significant using both Fisher’s exact test (p = 0.64) and *G* test (*G* = 0.221, p = 0.63). Thus, there is no evidence that the mitochondrial data are experiencing natural selection that might obscure phylogeographic signal. The Vaca and Telsen populations clustered with the outgroup taxa and are more genetically distant from the remaining ingroup samples (Table 2: and see nuclear phylogeny below). These individuals are therefore treated as an unrecognized taxon (new species) and were not included in subsequent analyses. Their distinctive and non-sister phylogenetic position suggests these populations are a distinct species from other aeglids. These populations are currently being described as a new species (Jara in prep). All remaining *A. neuquensis* samples formed the basal clade. Three main clades are found within this *A. neuquensis* clade. Two of these clades contain all the Negro and Neuquen individuals. Samples from Covunco, Bocatoma, Chimpay and El Alamito form a clade sister to a clade containing all the Ingeniero Ballester individuals. Samples from Cullin are shared between these two sister clades. The remaining samples from the Limay, Chubut and Chico systems form the remaining clade. Within this clade individuals from the Limay system are basal. Individuals from Chubut and Chico rivers are derived, monophyletic and sister to three haplotypes from the Pichileufu population in the upper reaches of the Limay River. The concatenated nDNA tree recovered three well-supported clades. One clade contained all the individuals from the Limay, Negro, and Neuquen rivers. There is relatively little support for relationships within this clade. As in the mitochondrial tree topology, all Vaca and Telsen are monophyletic and genetically distant from the remaining samples. All the Chico and Chubut haplotypes form the third clade and are sister, but with no support, to the Vaca and Telsen clade.

**Table 2:** Net divergence between Telsa + Vaca samples, outgroup and ingroup samples.

Locus	Grouping	Telsen+Vaca	Ingroup
mtDNA			
	Telsen+Vaca	–	
	Ingroup	0.042	–
	Outgroup	0.031	0.017
ANT Intron			
	Telsen+Vaca	–	
	Ingroup	0.006	–
	Outgroup	0.0	0.006
EF1 Intron			
	Telsen+Vaca	–	
	Ingroup	0.011	–
	Outgroup	0.007	0.011
EF1 Exon			
	Telsen+Vaca	–	
	Ingroup	0.008	–
	Outgroup	0.007	0.002
nuDNA Combined			
	Telsen+Vaca	–	
	Ingroup	0.008	–
	Outgroup	0.005	0.006

Between groups net means were computed using the Maximum Composite model with gamma distribution implemented in MEGA 5 [73].

The mitochondrial TMRCA for all *A. neuquensis* (minus Vaca and Telsen samples as they are a distinct species) was 235 ka (149–322 ka; Fig. 2). This range overlaps two glacial cycles, the last Patagonian glaciation (∼180 ka) and the previous cycle (∼270 ka). The mitochondrial TMRCA for the clade containing nearly all the Negro and Neuquensis river individuals was 219 ka (140–300 ka; Fig. 2). The youngest mitochondrial TMRCA is 29 ka (17–41 ka: Chico-Chubut clade, Fig. 2), which is consistent with the LGM (∼20 ka). See EBSP results for the TMRCA from the combined data sets.

### Networks

**Figure 4:**
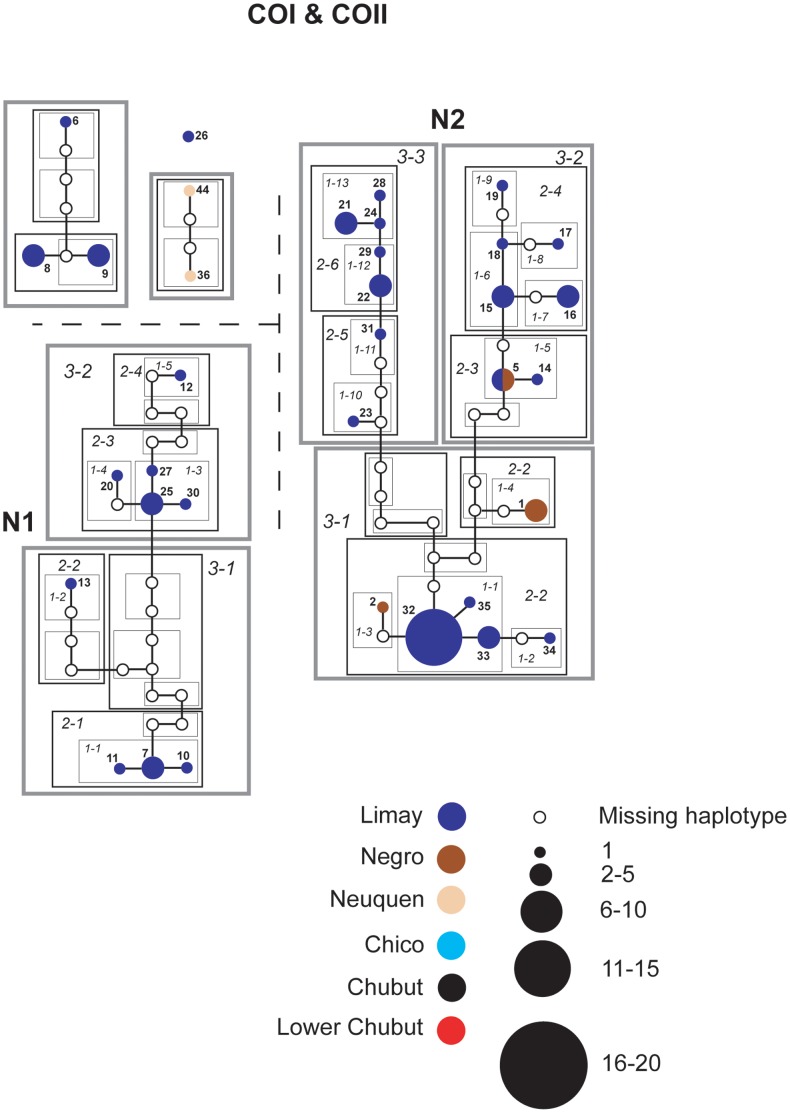
Haplotype networks with nested clade design for mtDNA (COI & COII) sequence data of Limay, Negro and Neuquen Rivers. Haplotype circle size is proportional to its frequency. Each haplotypes is numbered. Haplotypes that exceed twenty individuals are labeled with the total number of individuals of that haplotype. Arrow indicates the root haplotype. Black empty circles represent extinct (or unsampled) haplotypes. Color codes indicate the six major rivers.

**Figure 5:**
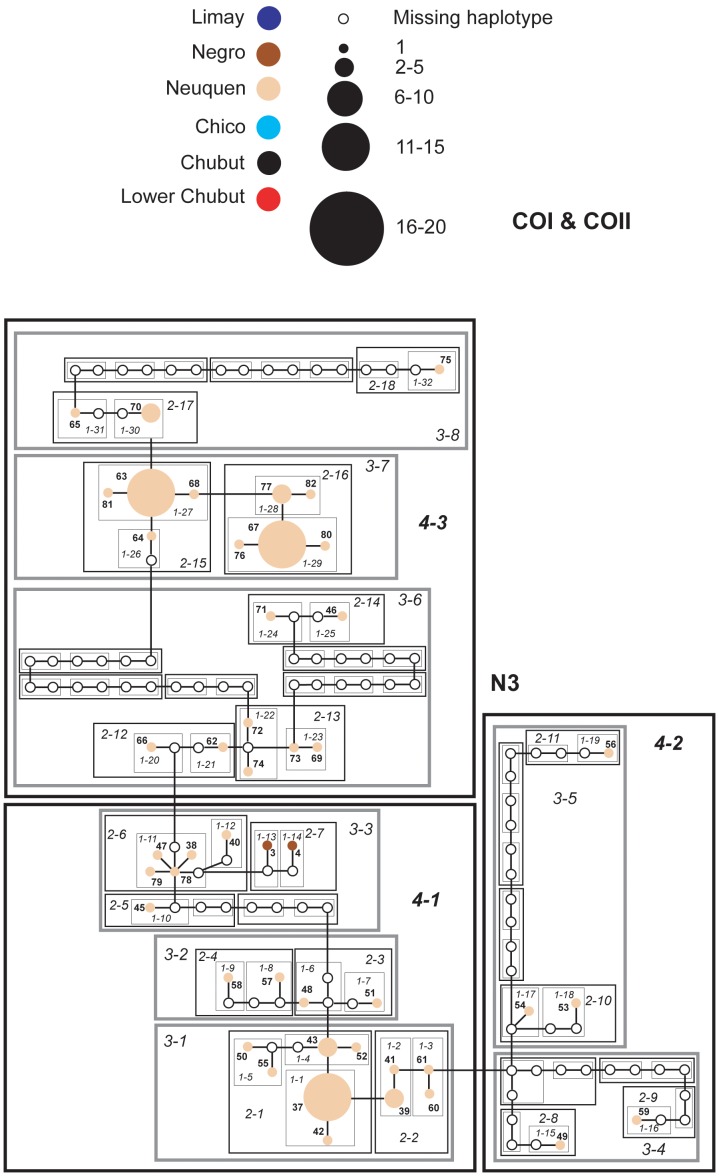
Haplotype networks with nested clade design for mtDNA (COI & COII) sequence data comprised mostly of individuals from the Neuquen River. Haplotype circle size is proportional to its frequency. Each haplotypes is numbered. Haplotypes that exceed twenty individuals are labeled with the total number of individuals of that haplotype. Arrow indicates the root haplotype. Black empty circles represent extinct (or unsampled) haplotypes. Color codes indicate the six major rivers.

**Figure 6:**
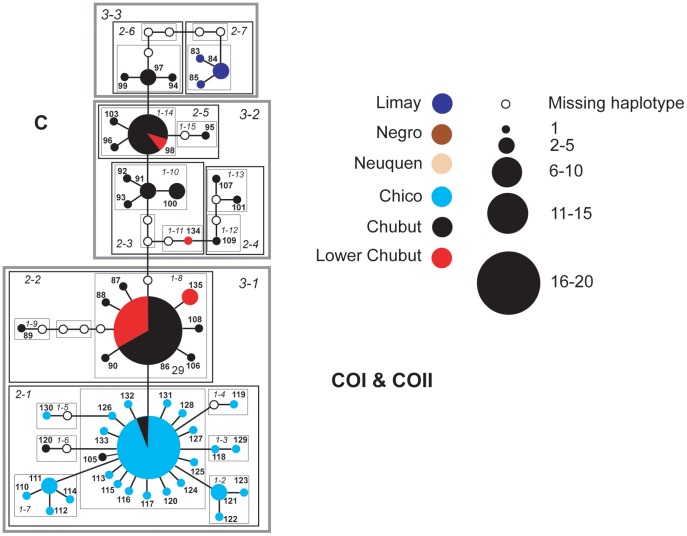
Haplotype network with nested clade design for mtDNA (COI & COII) sequence data comprised mostly of individuals from the Chico and Chubut Rivers. Haplotype circle size is proportional to its frequency. Each haplotypes is numbered. Haplotypes that exceed twenty individuals are labeled with the total number of individuals of that haplotype. Arrow indicates the root haplotype. Black empty circles represent extinct (or unsampled) haplotypes. Color codes indicate the six major rivers.

**Figure 7:**
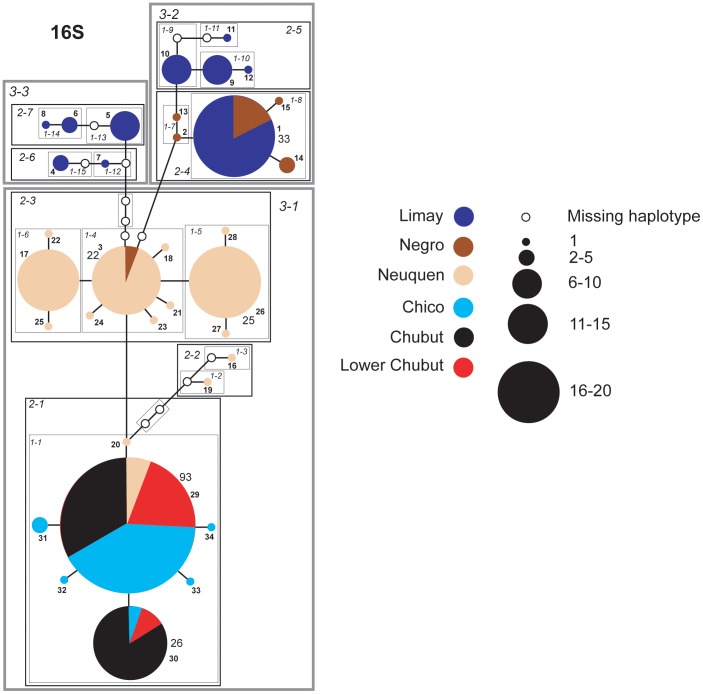
Haplotype network with nested clade design for mtDNA (16 S) sequence data comprised of all samples. Haplotype circle size is proportional to its frequency. Each haplotypes is numbered. Haplotypes that exceed twenty individuals are labeled with the total number of individuals of that haplotype. Arrow indicates the root haplotype. Black empty circles represent extinct (or unsampled) haplotypes. Color codes indicate the six major rivers.

**Figure 8:**
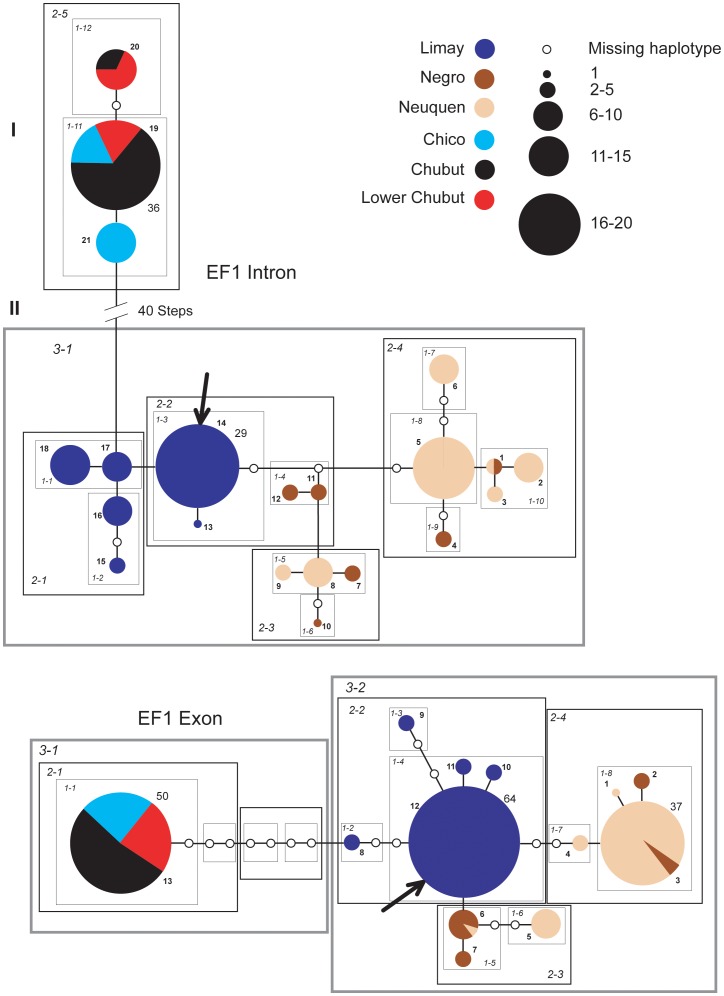
Haplotype networks with nested clade design for the nuclear EF1α intron and exon. Haplotype circle size is proportional to its frequency. Each haplotypes is numbered. Haplotypes that exceed twenty individuals are labeled with the total number of individuals of that haplotype. Arrow indicates the root haplotype. Black empty circles represent extinct (or unsampled) haplotypes. Color codes indicate the six major rivers.

**Figure 9:**
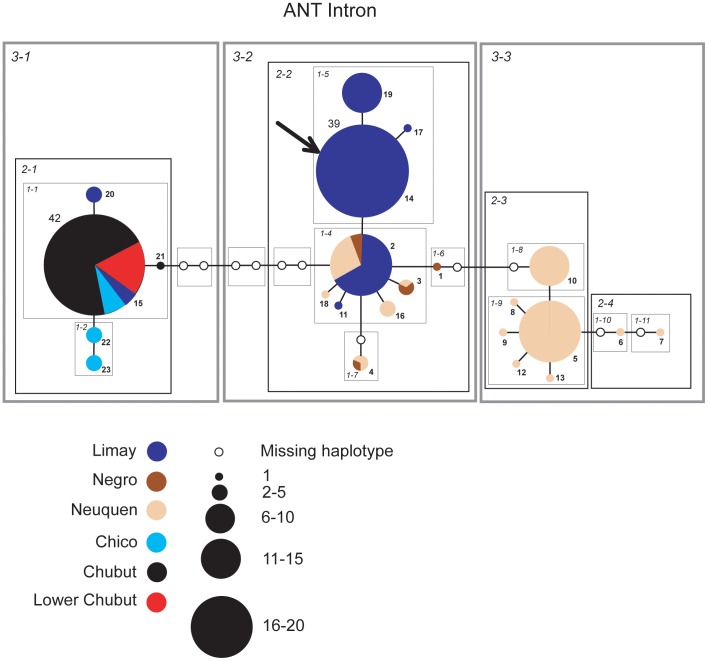
Haplotype networks with nested clade design for the nuclear ANT intron data. Haplotype circle size is proportional to its frequency. Each haplotypes is numbered. Haplotypes that exceed twenty individuals are labeled with the total number of individuals of that haplotype. Arrow indicates the root haplotype. Black empty circles represent extinct (or unsampled) haplotypes. Color codes indicate the six major rivers.

Individual haplotype networks from both genomes (Fig. 4, 5, 6, 7, 8, 9) show relationships that are consistent with those recovered in the phylogenetic analyses (Figs. 2, 3); that is, haplotypes from the Neuquen, Negro and Limay rivers are more closely related to one another than to those found in the Chubut-Chico systems. The ANT intron network is an exception because it has one widespread haplotype (#15) that is shared between these two systems and a Limay river haplotype (#20) that is more closely related to Chico-Chubut than it is to the other Negro system haplotypes (Fig. 9). Overall, networks group geographically close haplotypes. Nuclear networks further illustrate the relatively low haplotype diversity observed within the Chico-Chubut river system.

### Multilocus Nested Clade Phylogeographic Analysis

While NCPA found multiple significant inferences (Table 3), only three were detected across two or more loci (Table 4). Only these three will be considered for further discussion. Fragmentation (either past-fragmentation [PF] or allopatric-fragmentation [AF]) between the Limay and Chubut rivers was inferred in all four loci. As predicted this fragmentation event occurred between the headwaters of the Limay and Chubut rivers near the base of the Andes (Fig. 1). Restricted gene flow (RGF) was detected in the Chubut (three loci: ANT Intron, EF1 Exon, EF1 Intron) as well as in the Neuquen/Negro River (two loci: mtDNA and ANT Intron) systems.

**Table 3:** Nested Clade Phylogeographic Analysis [28], [29].

Clade	Chi-square	Probability	Inference chain	Inferred population process
**mtDNA**				
N1 Total	10.37	0.0029	1-2-11-17-No	Inconclusive Outcome
N2 3-1	21.71	0	1-2-3-4-No	RGF with IBD
N2 Total	101.16	0	1-2-3-4-9-No	AF
N3 2-1	11.10	0.0029	1-2-3-5-15-16-18-No	IGS to discriminate between Fragmentation, RE, and IBD
N3 4-1	22.43	0.0012	1-2-11-17-4-No	RGF with IBD
N3 Total*	67.38	0	1-2-3-4-No	RGF with IBD
C 2-1	49.20	0.0001	1-2-3-4-No	RGF with IBD
C 3-1	83.26	0	1-2-11-12-13-Yes	LDC or PRE possibly coupled with SF or PF followed by RE
C 3-2	29.20	0	1-2-3-4-No	RGF with IBD
C 3-3*	9.00	0.0088	1-19-20-2-3-4-9-No	AF
C Total	158.60	0	1-2-11-12-No	CRE
**nDNA**				
**EF1Intron**				
1–11	17.50	0.01	1-2-3-5-6-No	To few clades to discriminate
2–1	8.40	0.005	1-2-3-4-Yes	RGF with IBD
1–5	10.00	0.042	1-2-11-17-No	Inconclusive Outcome
2–4	78.18	0.0001	1-2-3-5-6-7-Yes	RGF but with LDD
1–2	10.00	0.041	1-2-11-17-4-Yes	RGF with IBD
2–2	36.00	0.0001	1-19-20-No	IGS
3–1*	271.64	0.0001	1-2-3-5-6-13-Yes	LDC
Total*	164.00	0.0001	1-19-Yes	AF
**EF1Exon**				
1–4*	39.38	0.0001	1-2-3-5-6-7-Yes	RGF but with LDD
2–3	12.86	0.003	1-2-3-4-5-6-7-Yes	RGF but with LDD
3–2	248.61	0	1-2-3-5-15-16-18-No	IGS to discriminate between Fragmentation, RE, and IBD
1–8	22.94	0.003	1-2-11-12-No	CRE
Total*	190.00	0	1-2-3-5-15-Yes	PF and/or LDC
**ANT intron**				
1–1*	51.94	0.016	1-2-3-4-No	RGF with IBD
3–1*	23.23	0.001	1-2-3-5-15-Yes	PF and/or LDC
1–4	41.93	0.001	1-2-11-17-4-No	RGF with IBD
1–5*	24.69	0.0001	1-2-3-5-6-7-Yes	RGF but with LDD
3–2	86.05	0	1-2-3-5-6-13-Yes	LDC or PRE possibly coupled with SF or PF followed by RE

Nested contingency results based on 10000 permutations. Clades with significant geographic associations are shown (*P*<0.05). Abbreviations for the inferences: RGF, restricted gene flow; IBD, isolation by distance; AF, allopatric fragmentation; IGS, inadequate geographical sampling; RE, range expansion; LDC, long distance colonization; PRE, past range expansion; SF, subsequent fragmentation; PF, past fragmentation; CRE, contiguous range expansion; LDD, long distance dispersal. Asterisks denote inferences in common across two or more loci (see Table 4).

**Table 4:** Multilocus NCPA [28], [29] inferences shared across more than one loci.

Inference	mtDNA	ANT Intron	EF1 Exon	EF1 Intron
Fragmentation between Limay andChubut Rivers	Clade C 3–3	Clade 2–1	Total Cladogram	Total Cladogram
Restricted Gene Flow (RGF) in Neuquen/Negro Rivers	–	Clade 1–4	Clade 2–3	Clade 2–4
Restricted Gene Flow (RGF) inChubut River	Clade C 3–2	Clade 1–1	–	–

Clade in which inference occurred is listed for each locus. See Fig. 4 for nested networks.

### Demographic Model Selection

The highest ranked demographic model using Akaike Information Criterion (AIC) was ABADE (Tables 5 and 6: four additional models could not be rejected using chi-square with or without Bonferroni correction). This model had unequal rates of gene flow between the Chico-Chubut and Negro systems with gene flow (where *M* = *m/*μ) from the Chico-Chubut into the Negro system (0.0404) greater than the reverse (0.0118). The estimated ancestral (*qa*) population (where θ = 4 Neμ) size was the same as the Negro system (8.3701), while the Chico-Chubut has the smallest population size (1.8135). The time of population divergence between these two river systems was 137 ka. The geometric mean of the mutation rate across loci was 4.008839×10^−5^/combined loci/generation. The 95% posterior probability distribution (not shown) for time (*t*) does not include zero, supporting the hypothesis of historical isolation (i.e., fragmentation) with post isolation gene flow. Each of the three IMa runs converged on nearly identical parameter estimates and had relatively high ESS values (>50: values for *t* tended to be lower). Thus, we conclude that these analyses were well sampled and converging on correct values and that we were justified in combining the three runs for the model selection procedure.

**Table 5:** Examination of 16 demographic models for the *Aegla neuquensis* data using IMa [66].

Model	*t*	log(P)	*q*1	*q*2	*qa*	*m*1	*m*2	df	2LLR	p value
**ABCDD**	**5.416**	**0.4949**	**7.527**	**1.6866**	**17.2358**	**0.0198**	**0.0198**	**1**	**0.6418**	**p = 0.4230**
ABCD0	2.1142	−2.8008	6.8691	1.5418	16.4712	0.0889	0.0001	1	7.2331	p = 0.0071
**ABC0D**	**3.2417**	**0.0642**	**7.8487**	**1.7645**	**12.7467**	**0.0001**	**0.0511**	**1**	**1.5032**	**p = 0.2201**
ABC00	2.7521	−5.1727	8.5806	1.9706	12.6997	0.0001	0.0001	2	11.9768	p = 0.0025
AACDE	2.0638	−64.2833	5.2478	5.2478	21.9838	0.1136	0.0001	1	130.198	p<0.001
AAADE	1.6781	−74.0919	5.4746	5.4746	5.4746	0.0122	0.0606	2	149.8152	p<0.001
AACDD	2.0511	−67.5347	5.245	5.245	21.6617	0.0776	0.0776	2	136.7008	p<0.001
AAC00	3.2388	−72.6696	4.7527	4.7527	43.4875	0.0001	0.0001	3	146.9707	p<0.001
AAADD	1.6808	−75.0209	5.4737	5.4737	5.4737	0.0264	0.0264	3	151.6733	p<0.001
AAA00	4.7015	−86.0782	6.0031	6.0031	6.0031	0.0001	0.0001	4	173.7879	p<0.001
**ABADE**	**5.507**	**−0.0534**	**8.3701**	**1.8135**	**8.3701**	**0.0118**	**0.0404**	**1**	**1.7384**	**p = 0.1873**
**ABADD**	**5.7177**	**−0.3562**	**8.3161**	**1.8441**	**8.3161**	**0.02**	**0.02**	**2**	**2.344**	**p = 0.3097**
ABA00	3.2466	−5.6022	8.9966	1.983	8.9966	0.0001	0.0001	3	12.8358	p = 0.0050
ABBDE	15.955	−1.6391	7.4915	1.5942	1.5942	0.0139	0.0192	1	4.9097	p = 0.0267
**ABBDD**	**15.975**	**−1.6627**	**7.4911**	**1.5886**	**1.5886**	**0.0154**	**0.0154**	**2**	**4.957**	**p = 0.0838**
ABB00	9.994	−10.5794	9.2855	1.9159	1.9159	0.0001	0.0001	3	22.7903	p<0.001

Population *q*1 and *q*2 refer to the Negro and Chubut river systems respectively, whereas *qa* is the ancestral population for the given model. Migration estimates are for gene flow from Negro into the Chubut system (*m*1) whereas *m*2 is the reverse direction. Gene flow estimates of 0.001 are effectively zero. Values for *t* and the five demographic parameters are the high points of the posterior distribution. Five models (in bold) could not be rejected at α = 0.05 or using a Bonferroni corrected α = 0.003125. Time of divergence (*t*) is not included in the model selection.

**Table 6:** Demographic models from Table 5 (plus Full Model) ranked using Akaike Information Criterion (AIC).

Model	log(P)	*k*	AIC	*t*	Divergence
**ABADE**	**−0.0534**	3	6.1068	**5.507**	137,000
**ABADD**	**−0.3562**	3	6.7124	**5.7177**	143,000
**ABCDD**	**0.4949**	4	7.0102	**5.416**	135,000
**ABBDD**	**−1.6627**	2	7.3254	**15.975**	398,000
**ABC0D**	**0.0642**	4	7.8716	**3.2417**	81,000
Full model	0.8158	5	8.3684	2.097	52,000
ABBDE	−1.6391	3	9.2782	15.955	398,000
ABCD0	−2.8008	4	13.6016	2.1142	53,000
ABA00	−5.6022	2	15.2044	3.2466	81,000
ABC00	−5.1727	3	16.3454	2.7521	67,000
ABB00	−10.5794	1	23.1588	9.994	250,000
AACDE	−64.2833	4	136.5666	2.0638	51,000
AACDD	−67.5347	3	141.0694	2.0511	51,000
AAC00	−72.6696	2	149.3392	3.2388	81,000
AAADD	−75.0209	2	154.0418	1.6808	42,000
AAADE	−74.0919	4	156.1838	1.6781	42,000
AAA00	−86.0782	3	178.1564	4.7015	117,000

AIC calculated using -2(log) +2*k,* (where *k* is the number of free parameters in the model). Models in bold as in table 5. Based on AIC score the model (ABADE) provides the best fit to our data. This model had equal population sizes between the Negro and ancestral populations and unequal gene flow between Negro and Chubut systems (Table 5). Estimates of the time of divergence (years before present [to the nearest thousand]) between river systems were obtained by dividing *t* by the geometric mean of the mutation rate across loci ( = 4.008839×10^−5^: see methods for further details).

### Gene Flow

**Figure 10:**
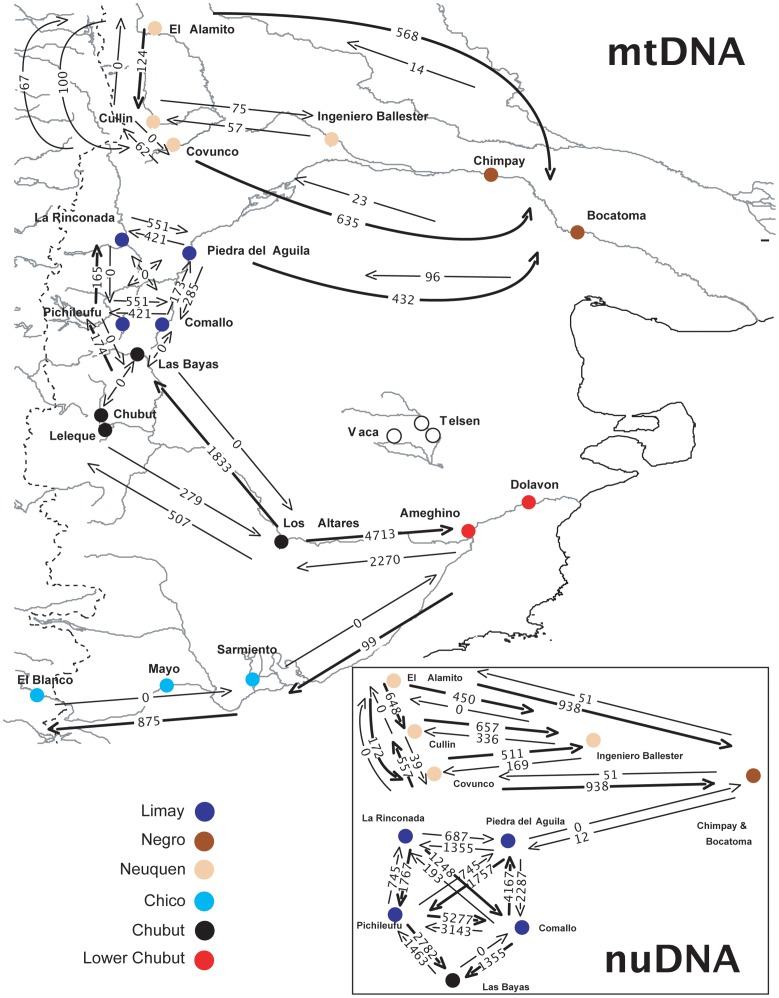
Gene flow estimates for mtDNA and a combined nDNA analysis using MIGRATE [71]. A thick arrow indicates gene flow that is at least two-times greater in one direction than the opposite direction. Color codes indicate the six major rivers.

Asymmetrical gene flow was observed in both mtDNA and nDNA with a greater magnitude of downstream flow detected within the Neuquen and Negro river system (Fig. 10). Similar patterns between mtDNA and nDNA suggests that there is no sex biased gene flow. Both mtDNA and nDNA suggest that the majority of populations in the upper reaches of the Limay River are exchanging genes. In the Chubut and Chico rivers, only mtDNA possessed sufficient variation to provide reliable estimates. In the Chubut system mitochondrial gene flow is occurring both upstream and downstream from the Los Altares population. Conversely, in the Chico River all gene flow is following an upstream pattern with no detectable flow occurring downstream. Both the Pichileufu and Comallo populations in the Limay River have exchanged nDNA with the Las Bayas population of the Chico River. In contrast, only the Pichileufu population has experienced mtDNA gene flow from Last Bayas and this gene flow was asymmetrical with Pichileufu receiving immigrants from Las Bayas.

### Extended Bayesian Skyline Plots

**Figure 11:**
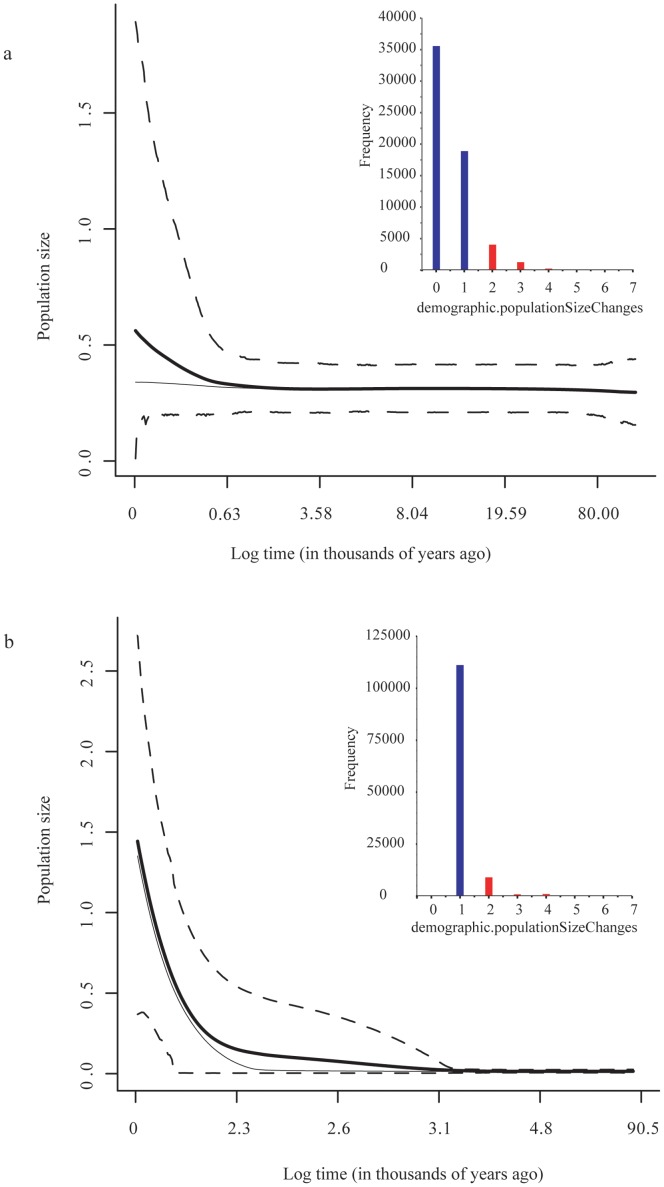
Extended Bayesian skyline plots of historical demographics of the two major river systems [(a) Negro system and (b) Chico-Chubut)] within *Aegla neuquensis*. The TMRCA is the mean root height. The median estimate (bold line), mean (light line) and 95% HPD limits are dashed. Inset is the number of demographic changes detected for each analysis. Blue bars indicated number of demographic changes that are within the 95% HPD estimates. Red bars are outside the 95% HPD.

Contrasting demographic histories between the two major river systems were estimated using EBSP. We cannot reject a constant demographic model for the Negro system (Fig. 11a inset). However, the 95% HPD includes one demographic change. Inspection of the EBSP suggests that this event could have occurred over the last 65 ka (Fig. 6a). In contrast, we can reject a constant demographic model for the Chico-Chubut system because the mode number of changes is one (Fig. 11b inset). The EBSP shows an explosive increase over the last 2–3 thousand years (Fig. 11b). These plots show that the Negro system’s TMRCA is much older than that of the Chico-Chubut, 300 ka and 48 ka, respectively. The effective population size for Chico-Chubut was much lower (near zero) than the Negro’s for most of the history of this population. Each of the three EBSP runs conducted for each system converged on similar estimates for our parameters of interest and these parameters had high (>200) ESS values. See the appendix for the BEAST *xml* files and plots of TMRCA for each locus.

### Population Genetic Analysis

Population genetic diversity as indicated by haplotype diversity (*Hd*) was generally high except those of Bayas, Covunco and Chubut, but nucleotide diversity (θ_π_) was relatively low, especially in the populations from the Chico-Chubut River (Table 1). These observations indicate a higher genetic diversity and deeper genetic divergence in populations from the Negro system than those from the Chico-Chubut system, results consistent with the phylogenies and networks. Stronger genetic divergence was observed between *A. neuquensis* populations (*Ф_ST_* = 0.774, *P* = 0; AMOVA test), and pairwise *Ф_ST_* values were large and significant (*P*<0.05) among all populations, excluding those between Bocatoma and Chimpay, Bocatoma and Cullin, Bocatoma and Pichileufu, Piedra del Aguila and La Rinconada, and Los Altares and Ameghino (Table 7). Genetic differentiations between the Neuquen-Negro River and Chico-Chubut River populations (*Ф_ST_* = 0.708−0.988) were the largest, followed by those between the Neuquen-Negro River and Limay River (0.503–0.986), while those between the Limay River and Chico-Chubut River (0.566–0.927) were relatively small. Total genetic diversity as measured by theta was much greater in Negro system versus that found in the Chico-Chubut system (Table 8).

**Table 7:** Pairwise *Ф_ST_* values (underlined are significant after sequential Bonferroni correction (*P*<0.05)) between populations of *A. neuquensis*.

Boc		Chi	InB	Ala	Cul	Cov	PiA	Rin	Com	Pic	Bay	Chu	Lel	Alt	Bla	May	Sar
Chi	−0.01																
InB	0.622	0.533															
Ala	0.622	0.382	0.732														
Cul	0.238	0.282	0.486	0.412													
Cov	0.715	0.532	0.785	0.841	0.447												
PiA	0.714	0.503	0.712	0.785	0.685	0.821											
Rin	0.854	0.643	0.788	0.892	0.755	0.918	0.093										
Com	0.706	0.544	0.688	0.759	0.663	0.775	0.424	0.524									
Pic	0.954	0.642	0.777	0.957	0.753	0.986	0.551	0.729	0.624								
Bay	0.976	0.829	0.87	0.972	0.837	0.985	0.669	0.825	0.72	0.927							
Chu	0.967	0.708	0.812	0.965	0.786	0.988	0.566	0.747	0.662	0.922	0.843						
Lel	0.963	0.764	0.835	0.963	0.805	0.982	0.6	0.766	0.673	0.845	0.838	0.504					
Alt	0.914	0.742	0.824	0.932	0.796	0.953	0.59	0.73	0.669	0.665	0.162	0.378	0.445				
Bla	0.966	0.803	0.856	0.965	0.823	0.98	0.641	0.8	0.701	0.881	0.517	0.815	0.756	0.248			
May	0.963	0.777	0.842	0.963	0.81	0.981	0.622	0.788	0.692	0.883	0.535	0.823	0.761	0.248	0.18		
Sar	0.96	0.793	0.85	0.962	0.819	0.977	0.635	0.792	0.699	0.858	0.464	0.789	0.732	0.218	0.083	0.098	
Ame	0.954	0.796	0.853	0.958	0.822	0.973	0.636	0.787	0.697	0.828	0.117	0.603	0.67	0.038	0.408	0.413	0.37

**Table 8:** Mitochondrial DNA diversity and neutrality test for *A. neuquensis*.

Region	*N*	*Hd*	θ_π_	θ_W_	Fu’s *Fs*	Tajima’s *D*
Limay-Neuquen-Negro River	155	0.979	0.0192	0.0167	−10.985**	0.0157
Chico-Chubut River	123	0.912	0.0019	0.0049	−52.084**	−1.973*

Sample size (*N*), haplotype diversity (*Hd*), current (θ_π_) and historical (θ_W_) genetic diversity; and Fu’s *Fs* and Tajima’s *D* test are shown. *P<0.05, **P<0.01.

Tajima’s D tests were significant (*P*<0.05) and negative for Bayas, Sarmiento (not shown) and Chico-Chubut River (Table 8). Fu’s *Fs* was significant and negative for Rinconada, Los Altares, El Blanco, Mayo, Sarmiento (not shown) and Negro and Chico-Chubut systems (Table 8). Significant and negative values of Tajima’s D and Fu’s *Fs* suggest that these populations and rivers have experienced recent demographic growth.

## Discussion

### Fragmentation Event

We find considerable support for our primary hypothesis of gene flow and fragmentation between the Negro and Chico-Chubut systems. These two systems are reciprocally monophyletic in our nuclear phylogeny. Evolutionary relationships primarily from genetic networks suggest that this fragmentation event and the between systems gene flow occurred between populations within the headwaters region of these two large river systems. Process inference from NCPA and an isolation-with-migration analysis provide corroboration for our primary hypothesis and additional details on the demographic history of this event. For example, multilocus NCPA analysis inferred this fragmentation event in all four loci. The highest-ranking demographic isolation-with-migration model suggests that the ancestral (pre-fragmentation) population was equal in size to the current size of the Negro population and that both are larger than the Chico-Chubut population. These relative population sizes are consistent with the patterns of haplotype diversity. The isolation-with-migration model suggests that gene flow between systems is asymmetrical, with up to four times as many individuals moving into the Chico-Chubut system from the Negro system as occurred in the opposite direction.

The fragmentation event between the Negro and Chico-Chubut systems occurred roughly 137 ka. Of the five major climatic events outlined by Ruzzante *et al.* [17], [19], only the Patagonian glaciation (∼180 ka) is consistent with our estimate. We posit that a paleolake formed in the headwaters region of these two systems during the Patagonian glaciation [16], [18]. The existence of a lake in this region is supported by the detection of limited gene flow between populations in the headwaters. Our isolation-with-migration analysis confirms post-isolation gene flow. Flooding of the region during the Patagonian glaciation would have provided opportunities for gene flow between systems. These two systems were then isolated again following the retreat of glaciers in the region. We find no evidence that these two systems came into contact in the deltoic mosaic that would have been present on the exposed continental shelf during the last glacial maximum [16].

Our estimate of the timing of this fragmentation event could be much older than 137 ka because mitochondrial introgression (see below) occurred after the two systems and the three nuclear loci diverged. Therefore our estimate of 137 ka could be biased when we combined these two genomes into a single analysis. Observation of reciprocal monophyly in nuclear loci over a relatively recent period further suggests that our divergence estimate is biased by mitochondrial introgression, however, the extremely low historical effective population size (a population bottleneck) observed in the Chico-Chubut system suggests that monophyly could have occurred over short evolutionary time.

Samples from the Vaca and Telsen Rivers are morphologically similar to *Aegla neuquensis* but are in fact more closely related to other *Aegla* taxa. These rivers are geographically isolated from other systems and have not been connected to rivers that contain other *A. neuquensis*. This evolutionarily independent group was treated as a new taxon in our analyses and will be formally described in a forthcoming work.

### Post Fragmentation History

Post fragmentation histories of these two systems were different. First, the Negro system appears to have been demographically stable and has maintained a greater amount of genetic diversity throughout its history. In contrast, the Chico-Chubut system has relatively lower levels of diversity even after a period of demographic expansion over the last several thousand years. In addition, the most recent common ancestor is younger in the Chico-Chubut system than in the Negro system.

Gene flow recovered using MIGRATE suggests very different patterns in these two systems and within individual rivers. Downstream gene flow in both mitochondrial and nuclear markers is extensive within most of the Negro system, with very little movement upstream. Conversely, gene flow is bidirectional between populations in the headwaters of the Limay River. Gene flow in the Chico River is exclusively upstream. Bidirectional flow is seen in the Chubut River, with the Los Altares population serving as the primary source of emigrants within this river. Both systems exhibited restricted gene flow. Differences in gene flow appear consistent with the overall patterns of genetic diversity and demographic history, in the following manner. First, the stable demographic history and larger genetic diversity of the Negro system is characteristic of populations at equilibrium and therefore not colonizing new habitats [34]. These observations would suggest that both females and males are being carried downstream within this system and that very few individuals are emigrating out of the Negro River upstream into the Limay or Neuquen Rivers. In stark contrast, the large amounts of upstream movement detected in the Chico-Chubut system especially in the Chico River is indicative of recent colonization, an inference consistent with the observation of low genetic diversity and recent demographic expansion.

The best demographic model detected some gene flow between systems after they were fragmented. This gene flow would have occurred within the last 137 ka. Only during the last glacial maximum (23–25 ka) would there have been the opportunity for individuals to move between systems via another paleolake. Our best model suggests that the majority of individuals moved south from the headwaters of the Neuquen River into the Chico River, though some appear to have dispersed in the reverse direction.

### Comparative Patterns and Processes Between Cis and Trans Andean *Aegla* Taxa

Patagonian freshwater systems are unique and geographically diverse. Andean orogeny (16–23 million years ago [Ma]) played a major role in shaping these systems primarily due to the mountain range’s relative proximity to Pacific and Atlantic coasts (Fig. 1: [35]). Pacific systems are short in length (∼200 km) and fast-flowing due to steep elevational gradients [36]. In comparison, Atlantic drainages are longer slow-moving rivers that are geographically distant from one another. Glacial cycles likewise affected Pacific and Atlantic river systems [37]. The impact of glaciation cycles on the two regions was remarkably different [36], [38]. The LGM covered a significant portion of Pacific drainages, but only a small portion–primarily in the headwaters–of Atlantic drainages were covered. These differences in geology and glacial impacts suggest that freshwater organisms in these two regions experienced different evolutionary histories [8].

Glacial cycles and geography played fundamental but different roles in the evolutionary history of *A. neuquensis* (this paper) and *A. alacalufi* [24]. Overall phylogenetic, NCPA, and demographic analyses suggest that glacial cycles were key drivers in shaping the evolutionary history of *A. alacalufi* [24]. For example, six clades with deeper divergences and more genetic structuring were detected in non-glaciated island populations. A single-shallow clade was recovered in the continental-glaciated populations. Populations of *A. alacalufi* persisted in the relatively ice-free western islands, whereas much of the mainland distribution was recently colonized. Xu *et al.* [24] proposed a novel colonization route where individuals followed glacial waters flowing from higher to lower elevations. Remarkably, this relatively complex history was formed in a relatively short time period over the last two glacial cycles. Glacial cycles played a very different, but nonetheless important, role in shaping the evolutionary history of *A. neuquensis*. In this taxon, only a small portion of the total distribution was directly impacted by glacial cycles [9], [18]. Regardless, this limited exposure to the direct effects of glacial cycles dramatically shaped the evolutionary history of this taxon by fragmenting it into two (three including the Telsen+Vaca clade) lineages. Later, during the last-glacial maximum gene flow occurred when another paleolake formed in the headwaters of the two systems. Demographic expansion was experienced after the last-glacial maximum in the Chico-Chubut system.

### A Case of Mitochondrial Introgression

Mitochondrial loci have long been the standard marker for phylogenetic and phylogeographic analyses because they are more likely to recover recent evolutionary history [3]. In this study, we used both nDNA and mtDNA to provide independent lines of evidence on the evolutionary history of *Aegla neuquensis*. Nearly all phylogeographic studies that have employed both nuclear and mitochondrial data have observed greater resolution in mitochondrial loci, including relatively deeper levels of genetic divergence in the mitochondrial versus the nuclear loci [3]. In this study of *Aegla neuquensis,* the opposite pattern was observed, though within rivers the mitochondrial locus was more informative. Multiple analyses of independent nuclear loci suggest that populations of *Aegla neuquensis* in the Chico-Chubut system are evolutionarily independent from those in the Limay-Negro system. Phylogenetic analysis of our mitochondrial locus recovered a non-monophyletic Limay system with Chico-Chubut haplotypes embedded within it. Additionally, and again contrary to theoretical expectations, genetic diversity was greater (between Limay-Negro and Chico-Chubut) in the nuclear loci than in the mitochondrial locus. Why are there conflicting signals between genomes?

Several alternative explanations have been proposed to explain conflicting signals between genomes [39], [40]. First, because of the stochastic nature of the evolutionary process one might detect greater resolution in a nuclear locus over mitochondrial simply by chance [3]. We reject this hypothesis because it is unlikely that three independently sorting nuclear loci would, by chance, recover a stronger signal than a more rapidly evolving mitochondrial locus. Another possible explanation is that the mitochondrial genome is under selection [39]. However, we did not detect selection in the mitochondrial locus. Extremely high female, relative to male, dispersal could explain the differences in loci. But to our knowledge no data exist to suggest a sex-based dispersal bias in this or any *Aegla* taxon and the breeding system of *Aegla* suggests that dispersal would be much greater in males than in females. Furthermore, our gene flow analysis suggests similar local patterns of gene flow for both nuclear and mitochondrial data sets.

The most likely explanation for the contradictory signal between genomes is interspecific mtDNA introgression with restricted nuclear gene flow. This phenomenon of “mitochondrial introgression” is observed when the mitochondrial genome of one species occurs against a predominant nuclear background of another species [40], [41], [42]. During hybridization, each species’ mitochondrial genome was exposed to the genetic backgrounds of the alternate species, and one species’ mtDNA variant (here individuals from the Limay River) may have relatively higher fitness than individuals from the Chico-Chubut system, causing the displacement of mtDNA in the Chico-Chubut by samples from the Limay River [40]. For the nuclear genomes involved in hybridization, selection against nuclear gene introgression in backcross generations could maintain their divergence and distinctness. Because of recombination of nuclear genes, backcross offspring could have various degrees of nuclear genes from the two parental species. If the genes of a parental species have co-adapted for a long time, it is conceivable that backcross offspring having more genes from a single species are favored over those having a mixture [43], i.e., reduced fitness in backcross generations caused by disruption of co-adapted parental gene complexes would be expected [41], [44], [45].

### Conclusions

A detailed understanding of the patterns and processes that shaped the evolutionary history of *Aegla neuquensis* was possible because we employed multiple analytical methods and loci from both nuclear and mitochondrial genomes [46]. Multiple loci allowed us to detect a recent fragmentation event between two of the major river systems of the region. We suggest that this fragmentation event was driven by the disappearance of a paleolake that had formed at the headwaters of these two river systems. By comparing phylogenetic patterns between mitochondrial and nuclear loci, we identified a rare case of mitochondrial introgression. Our interpretation of the phylogeographic history would have been misleading without the addition of nuclear loci. Future study of aquatic taxa in these two systems will determine the taxonomic breadth of the impact of this fragmentation event.

## Materials and Methods

### Sampling and Sequence Data

A total of 295 specimens were collected during 2006–09 from 21 locations spanning the entire distribution of *A. neuquensis*, an area approximately 3.4×10^5^ km^2^ (Fig. 1 and Table 1). Specimens were caught by dipnet or hand. Gill or muscle tissues were removed for DNA analysis. Individuals were preserved in ethanol and deposited in the crustacean collections of the Monte L. Bean Life Science Museum at Brigham Young University and the arthropod collections of Universidad Austral de Chile. As invertebrates, no specific permits were required. Locations were not privately owned nor protected and this species is not endangered.

DNA extraction, PCR amplification, and sequencing of three mitochondrial genes (16 S, COI and COII) are described in a previous study [24]. We added to these three nuclear fragments – EF1α exon, EF1α intron and ANT intron – using a subset of samples (*N* = 103) that represents each locality and major branches of mtDNA phylogenetic tree. Nuclear fragments were amplified with newly designed primers EF1AX1 (5′ GCTGAGCGTGAACGTGGTATCAC 3′) and EF1XR (5′ GTTTGTGTTGACCAGAATAAAC 3′), primers EF1F4 and EF1XR0 [24], and primer DecapANTF [47] and the newly designed primer ANTir1 (5′GCCTCAAGAGACATTGACCTTTA 3′). Sequences were edited with Sequencher 4.8 (Gene Codes Corporation) and aligned with MAFFT 5.0 [48], [49]. All haplotypes were deposited in Dryad under http://dx.doi.org/10.5061/dryad.93js1254.

### Phylogenetic Analysis

Phylogenetic analyses for the mitochondrial and nuclear data sets were conducted separately using maximum-likelihood (ML) and Bayesian methods. The mitochondrial and nuclear data consisted of haplotypes of concatenated sequences from *A. neuquensis* and sequences of outgroup species (*Aegla ringueleti, A. septentrionalis, A. humahuaca, A. jujuyana, A. sanlorenzo, A. inercalata, A. scamosa, A. platensis, A. uruguayana,* [23]). The best-fit models of sequence evolution were determined using Akaike information criterion (AIC) as implemented in JModeltest [50]. ML analyses were performed using RAxML 7.0 [51], with 1000 bootstrap runs and searching for the best-scoring ML tree. Individual α-shape parameters, GTR-rates, and base frequencies were estimated and optimized for each partition. Bayesian analyses were performed in MrBayes 3.2 [52], using four independent runs with random start trees. We ran 10 million generations using four chains, with sampling frequency of one per 1000 for each chain. Results were visualized and checked with Tracer 1.5 [53] and a burn-in of 20% discarded. Divergence times (time to the most recent common ancestor - TMRCA) of major nodes were estimated with BEAST 1.6.1 [54]. Phylogenetic analyses of the mitochondrial data suggested that they might be under selection (see results). We used a McDonald-Kreitman test [55] as implemented in DnaSP 5.0 [56] to determine if the mitochondrial data were under selection.

### Multilocus Nested Clade Phylogeographic Analysis

Multilocus nested clade phylogeographic analysis (NCPA) was used to test for historical and demographic events that are geographically congruent across two or more loci [28], [29]. This method has been recently criticized for its high false-positive rates in simulation studies [57], [58]. In this study NCPA was used in conjunction with other approaches [59] to examine both patterns and processes of divergence across loci and geography [46]. We consider inferences drawn from multiple approaches as strong evidence. Furthermore only inferences found in two or more loci will be considered [28], [29]. Haplotype networks were constructed with TCS [60]. Associations between haplotypes and geography were tested with GEODIS [61]. Ambiguous connections or loops in the networks were resolved following the three criteria listed in Pfenninger and Posada [62]. We calculated distance along rivers instead of direct distance for geographical analysis [63]. To show all relationships in the nuclear loci, we set the connection limit to 40 steps for the nuclear data. We used the default 95% connection limit for the mtDNA. Allelic phase for nDNA loci for this analysis as well as the IMa and MIGRATE analyses (next sections) were determined using PHASE [64], [65] with default settings.

### Model Selection and Demographic History

Demographic history between Chubut (Chico and Chubut) and Negro (Limay and Neuquen) river systems, using nDNA and mtDNA, was examined using an isolation-with-migration analysis implemented in IMa [66]. This analysis was conducted in successive stages. First, exploratory analyses were performed to determine appropriate prior settings (i.e., posterior distributions were contained within the prior range) and the number of chains that gave consistent results across multiple runs. The priors, chains, and other starting conditions determined from exploratory analyses were: q1 = 5, −q2 = 5, –qA = 10, −m1 = 1, −m2 = 1, –t = 18, and –n = 3. Three independent analyses were then run for 72 hours using MCMC simulations to sample genealogies and obtain parameter estimates. Genealogies from these three analyses were then combined into a single data set. Next, we evaluated the relative fit of 16 nested-demographic models [67] using a portion ( = 20,000) of genealogies from the combined dataset using the likelihood option in IMa. Five population parameters are estimated in each model. These are: ancestral theta θ_A_, theta of descendent population one θ_1_, theta of descendent population two θ_2_, and two measures of gene flow (*m*_1_ and *m*_2_) between the two daughter populations. In addition, the time of the split between daughter populations were estimated using IMa. The best models were determined using a chi-square distribution with and without Bonferroni correction. Akaike Information Criterion (AIC) was also employed to rank the models (see [67] for further details). Time (*t*) was converted to years before present using a rate of 0.118 substitutions/site/million years that was estimated from the closely related *A. alacalufi* [24]. Mutation rates for each nuclear locus were obtained by multiplying the ratio of the mutation rate scalars (mtDNA/nDNA) estimated during the IMa runs to the mtDNA mutation rate [68]. The final estimates of divergence times were obtained by dividing *t* by the geometric mean of the mutation rate across loci per generation [68].

### Population Analysis

Population genetic diversities, as indicated by haplotype diversity (*Hd*), nucleotide diversity, current genetic diversity (θ_π_) and historical genetic diversity (θ_W_), were assessed using DnaSP 5.0 [56]. Genetic divergence between populations was estimated using *Ф_ST_* index and population structure was measured with an analysis of molecular variance (AMOVA), as implemented in [69]. Significance levels for multiple tests were adjusted with the sequential correction [70].

### Estimating Gene Flow

Patterns of gene flow between populations were determined using MIGRATE 3.2.6 [71], [72]. Estimates were obtained by averaging the mean values across three independent analyses. A single analysis consisted of 10 short chains with every 20 steps retained with a total of 1000 steps recorded. Then three long chains were run for 5 million generations, with every 100th steps recorded. Each long chain consisted of four adaptively heated chains determined using default settings. Each analysis was replicated four times with the final estimate averaged across replicates. By design we set the burnin to 10,000 steps. Due to relatively low sample sizes, four pairs of populations were grouped into single populations. These were Chimpay and Bocatoma; Chubut and Leleque; Ameghino and Dolavon; and the Mayo and Sarmiento populations. Due to low nDNA haplotype diversity in the Chico-Chubut system, all samples were grouped into a single population. The migration matrix was designed to allow the exchange of genes only between populations occurring in the same river system. The only exception to this assumption was the allowance of gene flow between populations at the headwaters of the Limay and Chico-Chubut systems.

### Extended Bayesian Skyline Plots

Historical demography of the Chico-Chubut and Limay-Neuquen-Negro river systems was examined using Extended Bayesian skyline plots (EBSP) as implemented in BEAST 1.6.1 and visualized in Tracer 1.5 [53]. These analyses employed all four loci. Each analysis was run for 50 million generations with every 1000 generations retained. The first 10 million generations (20%) were removed as burnin. Multiple runs were conducted on each data set to check for consistency. Final parameter estimates were obtained by combining three runs. We concluded the analyses were well sampled when three independent runs converged on similar values and had >100 ESS values for our focal parameters. The first of the three runs was used to generate a skyline plot. Analyses were run with a coalescent tree and relaxed uncorrelated lognormal clock priors. We employed the mtDNA mutation rate of 0.118 substitutions/site/million years [24] and allowed the analysis to estimate the rates, relative to the mtDNA, for each nuclear loci under a lognormal prior. Site models determined using jModeltest [50] for each of the four loci were HKY+I+G (mtDNA), JC+I (EF1 Exon), GTR+I (ANT), and HKY+I+G (EF1 Intron). We provide the Beast *xml* files (Data File S1 and S2) and TMRCA plots for each loci (Figure S1).

## Supporting Information

Figure S1 Plots of time of most-recent-common-ancestor for individual loci from three independent runs and from the three runs combined. Analyzed using Extended Bayesian skyline plots (EBSP) as implemented in BEAST 1.6.1 and visualized in Tracer 1.5. Each analysis was run for 50 million generations with every 1000 generations retained. The first 10 million generations (20%) were removed as burnin. Analyses were run with a coalescent tree and relaxed uncorrelated lognormal clock priors. We employed the mtDNA mutation rate of 0.118 substitutions/site/million years [17] and allowed the analysis to estimate the rates, relative to the mtDNA, for each nuclear loci under a lognormal prior. Site models determined using jModeltest [35] for each of the four loci were HKY + I + G (mtDNA), JC + I (EF1 Exon), GTR + I (ANT), and HKY + I + G (EF1 Intron). All ESS values were greater than 500.
(DOC)

Data File S1 Beast input file (.xml) for Extended Bayesian Skyline Plot analysis of the Negro River system.
(PDF)

Data File S2 Beast input file (.xml) for Extended Bayesian Skyline Plot analysis of the Chico-Chubut River system.
(PDF)

## References

[1] Avise JC (2000). Phylogeography: the history and formation of species. Cambridge, Mass.: Harvard University Press. viii, 447 p..

[2] Hickerson MJ, Carstens BC, Cavender-Bares J, Crandall KA, Graham CH, et al (2010). Phylogeography’s past, present, and future: 10 years after Avise, 2000.. Molecular Phylogenetics and Evolution.

[3] Zink RM, Barrowclough GF (2008). Mitochondrial DNA under siege in avian phylogeography.. Molecular Ecology.

[4] Edwards S, Bensch S (2009). Looking forwards or looking backwards in avian phylogeography? A comment on Zink and Barrowclough 2008.. Molecular Ecology 18: 2930–2933; discussion 2934–2936.

[5] Barrowclough GF, Zink RM (2009). Funds enough, and time: mtDNA, nuDNA and the discovery of divergence.. Molecular Ecology.

[6] Brito HP, Edwards, V S (2009). Multilocus phylogeography and phylogenetics using sequence-based markers.. Genetica.

[7] Edwards SV, Beerli P (2000). Perspective: gene divergence, population divergence, and the variance in coalescence time in phylogeographic studies.. Evolution.

[8] Pérez-Losada M, Xu J, Jara CG, Crandall KA, Held C, Koenemann S, Schubart CD (2011). Comparing phylogeographic patterns across the Patagonian Andes in two freshwater crabs of the genus *Aegla* (Decapoda: Aeglidae)..

[9] Clapperton CM (1993). Quaternary geology and geomorphology of South America.. Amsterdam: Elsevier.

[10] Ruzzante DE, Walde SJ, Cussac VE, Dalebout ML, Seibert J (2006). Phylogeography of the Percichthyidae (Pisces) in Patagonia: roles of orogeny, glaciation, and volcanism.. Molecular Ecology.

[11] Ruzzante DE, Walde SJ, Gosse JC, Cussac VE, Habit E (2008). Climate control on ancestral population dynamics: insight from Patagonian fish phylogeography.. Molecular Ecology.

[12] Zattara EE, Premoli AC (2005). Genetic structuring in Andean landlocked populations of *Galaxias maculatus*: effects of biogeographic history.. Journal of Biogeography.

[13] Zemlak TS, Habit EM, Walde SJ, Battini MA, Adams ED (2008). Across the southern Andes on fin: glacial refugia, drainage reversals and a secondary contact zone revealed by the phylogeographical signal of *Galaxias platei* in Patagonia.. Molecular Ecology.

[14] Zemlak TS, Habit EM, Walde SJ, Carrea C, Ruzzante DE (2010). Surviving historical Patagonian landscapes and climate: molecular insights from *Galaxias maculatus*.. BMC Evolutionary Biology.

[15] Morrone J, Lopretto E (1994). Distributional patterns of freshwater Decapoda (Crustacea: Malacostraca) in Southern South America: a panbiogeographic approach.. Journal of Biogeography.

[16] Ponce JF, Rabassa J, Coronato A, Borromei AM (2011). Palaeogeographical evolution of the Atlantic coast of Pampa and Patagonia from the last glacial maximum to the Middle Holocene.. Biological Journal of the Linnean Society.

[17] Ruzzante DE, Walde SJ, Macchi PJ, Alonso M, Barriga JP (2011). Phylogeography and phenotypic diversification in the Patagonian fish Percichthys trucha: the roles of Quaternary glacial cycles and natural selection.. Biological Journal of the Linnean Society.

[18] Rabassa J, Clapperton CM (1990). Quaternary glaciations of the southern Andes.. Quaternary Science Reviews.

[19] Ruzzante DE, Walde SJ, Gosse JC, Cussac VE, Habit E (2008). Climate control on ancestral population dynamics: insight from Patagonian fish phylogeography.. Molecular Ecology.

[20] Singer BS, Ackert RP, Guillou H (2004). 40Ar/39Ar and K-Ar chronology of Pleistocene glaciations in Patagonia.. Geological Society of America Bulletin.

[21] Sugden DE, Bentley MJ, Fogwill CJ, Hulton NRJ, McCulloch RD (2005). Late-glacial glacial events in Southernmost South America: a blend of ‘Northern’ and ‘Southern’ hemisphere climatic signals?. Geografiska Annaler: Series A, Physical Geography.

[22] Zink RM, Klicka J, Barber BR (2004). The tempo of avian diversification during the Quaternary.. Philosophical Transactions of the Royal Society of London Series B-Biological Sciences.

[23] Perez-Losada M, Bond-Buckup G, Jara CG, Crandall KA (2004). Molecular systematics and biogeography of the southern South american freshwater “crabs” *Aegla* (decapoda: Anomura: Aeglidae) using multiple heuristic tree search approaches.. Systematic Biology.

[24] Xu J, Pérez-Losada M, Jara CG, Crandall KA (2009). Pleistocene glaciation leaves deep signature on the freshwater crab *Aegla alacalufi* in Chilean Patagonia.. Molecular Ecology.

[25] Pérez-Losada M, Bond-Buckup G, Jara CG, Crandall KA (2009). Conservation Assessment of Southern South American Freshwater Ecoregions on the Basis of the Distribution and Genetic Diversity of Crabs from the Genus *Aegla*.. Conservation Biology.

[26] Pérez-Losada M, Jara CG, Bond-Buckup G, Crandall KA (2002). Phylogenetic relationships among the species of *Aegla* (Anomura: Aeglidae) freshwater crabs from Chile.. Journal of Crustacean Biology.

[27] Pérez-Losada M, Jara CG, Bond-Buckup G, Crandall KA (2002). Conservation phylogenetics of Chilean freshwater crabs *Aegla* (Anomura, Aeglidae): assigning priorities for aquatic habitat protection.. Biological Conservation.

[28] Templeton AR (2002). Out of Africa again and again.. Nature.

[29] Templeton AR (2004). Statistical phylogeography: methods of evaluating and minimizing inference errors.. Molecular Ecology.

[30] Barber BR, Unmack PJ, Perez-Losada M, Johnson JB, Crandall KA (2011). Different processes lead to similar patterns: a test of codivergence and the role of sea level and climate changes in shaping a southern temperate freshwater assemblage.. Bmc Evolutionary Biology.

[31] Beheregaray LB (2008). Twenty years of phylogeography: the state of the field and the challenges for the Southern Hemisphere.. Molecular Ecology.

[32] Ruzzante DE, Rabassa J (2011). Palaeogeography and palaeoclimatology of Patagonia: effects on biodiversity.. Biological Journal of the Linnean Society.

[33] Sérsic AN, Cosacov A, Cocucci AA, Johnson LA, Pozner R (2011). Emerging phylogeographical patterns of plants and terrestrial vertebrates from Patagonia.. Biological Journal of the Linnean Society.

[34] Crandall KA, Templeton AR (1993). Empirical tests of some predictions from coalescence theory.. Genetics.

[35] Folguera A, Orts D, Spagnuolo M, Vera ER, Litvak V (2011). A review of Late Cretaceous to Quaternary palaeogeography of the southern Andes.. Biological Journal of the Linnean Society.

[36] Unmack PJ, Bennin AP, Habit EM, Victoriano PF, Johnson JB (2009). Impact of ocean barriers, topography, and glaciation on the phylogeography of the catfish *Trichomycterus areolatus* (Teleostei: Trichomycteridae) in Chile.. Biological Journal of the Linnean Society.

[37] McCulloch RD, Bentley MJ, Purves RS, Hulton NRJ, Sugden DE (2000). Climatic inferences from glacial and palaeoecological evidence at the last glacial termination, southern South America.. Journal of Quaternary Science.

[38] Rabassa J, Coronato A, Martinez O (2011). Late Cenozoic glaciations in Patagonia and Tierra del Fuego: an updated review.. Biological Journal of the Linnean Society.

[39] Ballard JWO, Whitlock MC (2004). The incomplete natural history of mitochondria.. Molecular Ecology.

[40] Shaw KL (2002). Conflict between nuclear and mitochondrial DNA phylogenies of a recent species radiation: what mtDNA reveals and conceals about modes of speciation in Hawaiian crickets.. Proceedings of the National Academy of Sciences.

[41] Avise JC (2004). Molecular markers, natural history, and evolution. Sunderland, Mass.: Sinauer Associates.. 684 p.

[42] Perry WL, Feder JL, Dwyer G, Lodge DM (2001). Hybrid zone dynamics and species replacement between *Orconectes crayfishes* in a Northern Wisconsin lake.. Evolution.

[43] Powell JR (1983). Interspecific cytoplasmic gene flow in the absence of nuclear gene flow: evidence from *Drosophila*.. Proceedings of the National Academy of Sciences.

[44] Grainger Hunt W, Selander RK (1973). Biochemical genetics of hybridisation in european house mice.. Heredity.

[45] Carson HL, Templeton AR (1984). Genetic revolutions in relation to speciation phenomena: the founding of new populations.. Annual Review of Ecology and Systematics.

[46] Johnson JB, Crandall KA (2009). Expanding the toolbox for phylogeographic analysis.. Molecular Ecology.

[47] Teske PR, Beheregaray LB (2009). Intron-spanning primers for the amplification of the nuclear ANT gene in decapod crustaceans.. Molecular Ecology Resources.

[48] Katoh K, Kuma K-i, Toh H, Miyata T (2005). MAFFT version 5: improvement in accuracy of multiple sequence alignment.. Nucleic Acids Research.

[49] Katoh K, Toh H (2008). Recent developments in the MAFFT multiple sequence alignment program.. Briefings in Bioinformatics.

[50] Posada D (2008). jModelTest: Phylogenetic Model Averaging.. Molecular Biology and Evolution.

[51] Stamatakis A (2006). RAxML-VI-HPC: maximum likelihood-based phylogenetic analyses with thousands of taxa and mixed models.. Bioinformatics.

[52] Ronquist F, Huelsenbeck JP (2003). MrBayes 3: Bayesian phylogenetic inference under mixed models.. Bioinformatics.

[53] Rambaut A, Drummond AJ (2007). Tracer v1.4..

[54] Drummond A, Rambaut A (2007). BEAST: Bayesian evolutionary analysis by sampling trees.. Bmc Evolutionary Biology.

[55] John HM, Martin K (1991). Adaptive protein evolution at the Adh locus in *Drosophila*.. Nature Publishing Group.

[56] Librado P, Rozas J (2009). DnaSP v5: a software for comprehensive analysis of DNA polymorphism data.. Bioinformatics.

[57] Knowles LL (2008). Why does a method that fails continue to be used?. Evolution.

[58] Petit RJ (2008). The coup de grâce for the nested clade phylogeographic analysis?. Molecular Ecology.

[59] Garrick RC, Dyer RJ, Beheregaray LB, Sunnucks P (2008). Babies and bathwater: a comment on the premature obituary for nested clade phylogeographical analysis.. Molecular Ecology.

[60] Clement M, Posada D, Crandall KA (2000). TCS: a computer program to estimate gene genealogies.. Molecular Ecology.

[61] Posada D, Crandall KA, Templeton AR (2000). GeoDis: a program for the cladistic nested analysis of the geographical distribution of genetic haplotypes.. Molecular Ecology.

[62] Pfenninger M, Posada D (2002). Phylogeographic history of the land snail *Canidula unifasciata* (Helicellinae, Stylommatophoroa): fragmentation, corridor migration, and secondary contact.. Evolution.

[63] Fetzner JW, Crandall KA (2003). Linear habitats and the nested clade analysis: an empirical evaluation of geographic versus river distances using an Ozark crayfish (Decapoda: Cambaridae).. Evolution.

[64] Stephens M, Donnelly P (2003). A comparison of Bayesian methods for haplotype reconstruction from population genotype data.. American journal of human genetics.

[65] Stephens M, Smith NJ, Donnelly P (2001). A New Statistical Method for Haplotype Reconstruction from Population Data.. American journal of human genetics.

[66] Hey J, Nielsen R (2007). Integration within the Felsenstein equation for improved Markov chain Monte Carlo methods in population genetics.. Proceedings of the National Academy of Sciences.

[67] Carstens BC, Stoute HN, Reid NM (2009). An information-theoretical approach to phylogeography.. Molecular Ecology.

[68] Won Y-J, Hey J (2005). Divergence population genetics of chimpanzees.. Molecular Biology and Evolution.

[69] Laurent E, Guillaume L, Stefan S (2006). Arlequin ver 3.1– An Integrated Software Package for Population Genetics Data Analysis..

[70] Rice RW (1989). Analyzing tables of statistical tests.. Hoboken, NJ, ETATS-UNIS: Wiley.

[71] Beerli P, editor (2009). How to use migrate or why are markov chain monte carlo programs difficult to use? Cambridge: Cambridge University Press.. 42–79 p.

[72] Beerli P (2006). Comparison of Bayesian and maximum-likelihood inference of population genetic parameters.. Bioinformatics.

[73] Tamura K, Peterson D, Peterson N, Stecher G, Nei M, et al. MEGA5: Molecular Evolutionary Genetics Analysis Using Maximum Likelihood, Evolutionary Distance, and Maximum Parsimony Methods.. Molecular Biology and Evolution.

