# Supplementary figures and images for: Conflicting Evolutionary Patterns Due to Mitochondrial Introgression and Multilocus Phylogeography of the Patagonian Freshwater Crab Aegla neuquensis
Source: PLoS One. 2012 Jun 7;7(6):e37105. doi: 10.1371/journal.pone.0037105 (PMC3369872; doi:10.1371/journal.pone.0037105)

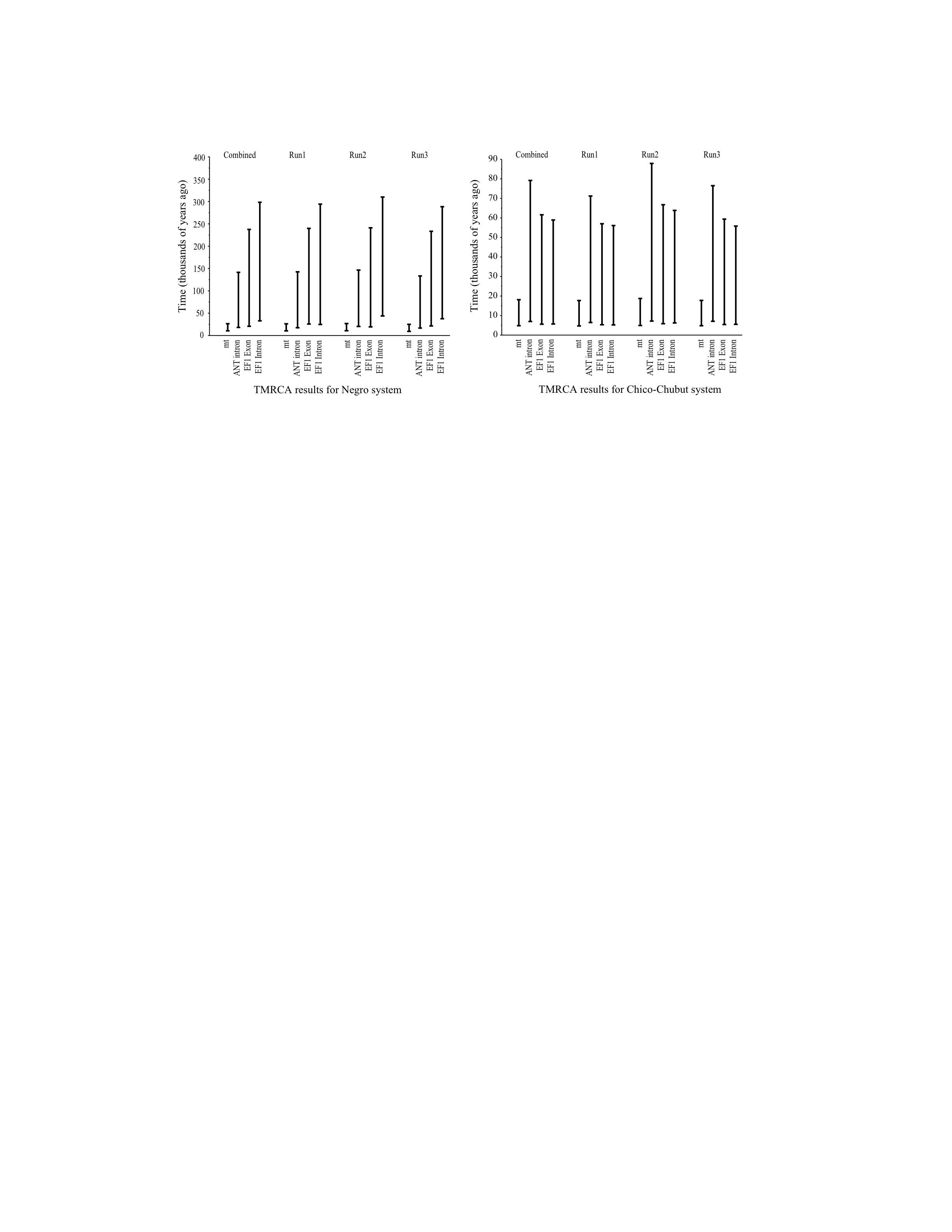

Supplement: Figure S1 — Plots of time of most-recent-common-ancestor for individual loci from three independent runs and from the three runs combined. Analyzed using Extended Bayesian skyline plots (EBSP) as implemented in BEAST 1.6.1 and visualized in Tracer 1.5. Each analysis was run for 50 million generations with every 1000 generations retained. The first 10 million generations (20%) were removed as burnin. Analyses were run with a coalescent tree and relaxed uncorrelated lognormal clock priors. We employed the mtDNA mutation rate of 0.118 substitutions/site/million years [17] and allowed the analysis to estimate the rates, relative to the mtDNA, for each nuclear loci under a lognormal prior. Site models determined using jModeltest [35] for each of the four loci were HKY + I + G (mtDNA), JC + I (EF1 Exon), GTR + I (ANT), and HKY + I + G (EF1 Intron). All ESS values were greater than 500. (DOC) [file pone.0037105.s001.doc]
