# Supplementary material for: Conflicting Evolutionary Patterns Due to Mitochondrial Introgression and Multilocus Phylogeography of the Patagonian Freshwater Crab Aegla neuquensis
Source: PLoS One. 2012 Jun 7;7(6):e37105. doi: 10.1371/journal.pone.0037105 (PMC3369872; doi:10.1371/journal.pone.0037105)
Supplement: Data File S1 — Beast input file (.xml) for Extended Bayesian Skyline Plot analysis of the Negro River system. (PDF) [file pone.0037105.s002.pdf]

```xml
<?xml version="1.0" standalone="yes"?>

<!-- Generated by BEAUTi v1.6.1                                              -->
<!--       by Alexei J. Drummond and Andrew Rambaut                          -->
<!--       Department of Computer Science, University of Auckland and        -->
<!--       Institute of Evolutionary Biology, University of Edinburgh        -->
<!--       http://beast.bio.ed.ac.uk/                                        -->
<beast>

    <!-- The list of taxa analyse (can also include dates/ages).                 -->
    <!-- ntax=413                                                                -->
    <taxa id="taxa">

*****DATA sections removed to reduce file size******

    </alignment>

    <!-- The unique patterns from 1 to end                                       -->
    <!-- npatterns=152                                                           -->
    <patterns id="A_neuq_mt_negro.patterns" from="1">
        <alignment idref="alignment1"/>
    </patterns>

    <!-- The unique patterns from 1 to end                                       -->
    <!-- npatterns=33                                                            -->
    <patterns id="A_neuq_nu_ANT_Negro.patterns" from="1">
        <alignment idref="alignment2"/>
    </patterns>

    <!-- The unique patterns from 1 to end                                       -->
    <!-- npatterns=26                                                            -->
    <patterns id="A_neuq_nu_EF1Exon_Negro.patterns" from="1">
        <alignment idref="alignment3"/>
    </patterns>

    <!-- The unique patterns from 1 to end                                       -->
    <!-- npatterns=37                                                            -->
    <patterns id="A_neuq_nu_EF1Intron_Negro.patterns" from="1">
        <alignment idref="alignment4"/>
    </patterns>

    <!-- This is a simple constant population size coalescent model              -->
    <!-- that is used to generate an initial tree for the chain.                 -->
    <constantSize id="initialDemo" units="substitutions">
        <populationSize>
            <parameter id="initialDemo.popSize" value="100.0"/>
        </populationSize>
    </constantSize>

    <!-- Construct a rough-and-ready UPGMA tree as an starting tree              -->
    <upgmaTree id="A_neuq_mt_negro.startingTree">
        <distanceMatrix correction="JC">
            <patterns>

                <!-- To generate UPGMA starting tree, only use the 1st aligment, which may be 1
                <alignment idref="alignment1"/>
            </patterns>
```

```
        </distanceMatrix>
    </upgmaTree>

    <!-- Construct a rough-and-ready UPGMA tree as an starting tree              -->
    <upgmaTree id="A_neuq_nu_ANT_Negro.startingTree">
        <distanceMatrix correction="JC">
            <patterns>

                <!-- To generate UPGMA starting tree, only use the 1st aligment, which may be 1
                <alignment idref="alignment2"/>
            </patterns>
        </distanceMatrix>
    </upgmaTree>

    <!-- Construct a rough-and-ready UPGMA tree as an starting tree              -->
    <upgmaTree id="A_neuq_nu_EF1Exon_Negro.startingTree">
        <distanceMatrix correction="JC">
            <patterns>

                <!-- To generate UPGMA starting tree, only use the 1st aligment, which may be 1
                <alignment idref="alignment3"/>
            </patterns>
        </distanceMatrix>
    </upgmaTree>

    <!-- Construct a rough-and-ready UPGMA tree as an starting tree              -->
    <upgmaTree id="A_neuq_nu_EF1Intron_Negro.startingTree">
        <distanceMatrix correction="JC">
            <patterns>

                <!-- To generate UPGMA starting tree, only use the 1st aligment, which may be 1
                <alignment idref="alignment4"/>
            </patterns>
        </distanceMatrix>
    </upgmaTree>

    <!-- Generate a tree model                                                   -->
    <treeModel id="A_neuq_mt_negro.treeModel">
        <upgmaTree idref="A_neuq_mt_negro.startingTree"/>
        <rootHeight>
            <parameter id="A_neuq_mt_negro.treeModel.rootHeight"/>
        </rootHeight>
        <nodeHeights internalNodes="true">
            <parameter id="A_neuq_mt_negro.treeModel.internalNodeHeights"/>
        </nodeHeights>
        <nodeHeights internalNodes="true" rootNode="true">
            <parameter id="A_neuq_mt_negro.treeModel.allInternalNodeHeights"/>
        </nodeHeights>
    </treeModel>

    <!-- Generate a tree model                                                   -->
    <treeModel id="A_neuq_nu_ANT_Negro.treeModel">
        <upgmaTree idref="A_neuq_nu_ANT_Negro.startingTree"/>
        <rootHeight>
            <parameter id="A_neuq_nu_ANT_Negro.treeModel.rootHeight"/>
        </rootHeight>
        <nodeHeights internalNodes="true">
```

```
            <parameter id="A_neuq_nu_ANT_Negro.treeModel.internalNodeHeights"/>
        </nodeHeights>
        <nodeHeights internalNodes="true" rootNode="true">
            <parameter id="A_neuq_nu_ANT_Negro.treeModel.allInternalNodeHeights"/>
        </nodeHeights>
    </treeModel>

    <!-- Generate a tree model                                                  -->
    <treeModel id="A_neuq_nu_EF1Exon_Negro.treeModel">
        <upgmaTree idref="A_neuq_nu_EF1Exon_Negro.startingTree"/>
        <rootHeight>
            <parameter id="A_neuq_nu_EF1Exon_Negro.treeModel.rootHeight"/>
        </rootHeight>
        <nodeHeights internalNodes="true">
            <parameter id="A_neuq_nu_EF1Exon_Negro.treeModel.internalNodeHeights"/>
        </nodeHeights>
        <nodeHeights internalNodes="true" rootNode="true">
            <parameter id="A_neuq_nu_EF1Exon_Negro.treeModel.allInternalNodeHeights"/>
        </nodeHeights>
    </treeModel>

    <!-- Generate a tree model                                                  -->
    <treeModel id="A_neuq_nu_EF1Intron_Negro.treeModel">
        <upgmaTree idref="A_neuq_nu_EF1Intron_Negro.startingTree"/>
        <rootHeight>
            <parameter id="A_neuq_nu_EF1Intron_Negro.treeModel.rootHeight"/>
        </rootHeight>
        <nodeHeights internalNodes="true">
            <parameter id="A_neuq_nu_EF1Intron_Negro.treeModel.internalNodeHeights"/>
        </nodeHeights>
        <nodeHeights internalNodes="true" rootNode="true">
            <parameter id="A_neuq_nu_EF1Intron_Negro.treeModel.allInternalNodeHeights"/>
        </nodeHeights>
    </treeModel>

    <!-- Generate a variableDemographic for extended Bayesian skyline process    -->
    <variableDemographic id="demographic" type="linear" useMidpoints="true">
        <populationSizes>

            <!-- popSize value = populationMean value                               -->
            <parameter id="demographic.popSize" value="1.0"/>
        </populationSizes>
        <indicators>
            <parameter id="demographic.indicators" value="0.0"/>
        </indicators>
        <trees>
            <ptree ploidy="0.5">
                <treeModel idref="A_neuq_mt_negro.treeModel"/>
            </ptree>
            <ptree ploidy="2.0">
                <treeModel idref="A_neuq_nu_ANT_Negro.treeModel"/>
            </ptree>
            <ptree ploidy="2.0">
                <treeModel idref="A_neuq_nu_EF1Exon_Negro.treeModel"/>
            </ptree>
            <ptree ploidy="2.0">
                <treeModel idref="A_neuq_nu_EF1Intron_Negro.treeModel"/>
```

```
            </ptree>
        </trees>
    </variableDemographic>
    <coalescentLikelihood id="coalescent">
        <model>
            <variableDemographic idref="demographic"/>
        </model>

        <!-- Take population Tree from demographic                                   -->
    </coalescentLikelihood>
    <sumStatistic id="demographic.populationSizeChanges" elementwise="true">
        <parameter idref="demographic.indicators"/>
    </sumStatistic>
    <exponentialDistributionModel id="demographic.populationMeanDist">
        <mean>

            <!-- prefer populationMean value = 1                                         -->
            <parameter id="demographic.populationMean" value="1.0"/>
        </mean>
    </exponentialDistributionModel>

    <!-- The uncorrelated relaxed clock (Drummond, Ho, Phillips & Rambaut, 2006) -->
    <discretizedBranchRates id="A_neuq_mt_negro.branchRates">
        <treeModel idref="A_neuq_mt_negro.treeModel"/>
        <distribution>
            <logNormalDistributionModel meanInRealSpace="true">
                <mean>
                    <parameter id="A_neuq_mt_negro.ucld.mean" value="0.118"/>
                </mean>
                <stdev>
                    <parameter id="A_neuq_mt_negro.ucld.stdev" value="0.3333333333333333" lower=
                </stdev>
            </logNormalDistributionModel>
        </distribution>
        <rateCategories>
            <parameter id="A_neuq_mt_negro.branchRates.categories" dimension="290"/>
        </rateCategories>
    </discretizedBranchRates>
    <rateStatistic id="A_neuq_mt_negro.meanRate" name="A_neuq_mt_negro.meanRate" mode="mean" int
        <treeModel idref="A_neuq_mt_negro.treeModel"/>
        <discretizedBranchRates idref="A_neuq_mt_negro.branchRates"/>
    </rateStatistic>
    <rateStatistic id="A_neuq_mt_negro.coefficientOfVariation" name="A_neuq_mt_negro.coefficient
        <treeModel idref="A_neuq_mt_negro.treeModel"/>
        <discretizedBranchRates idref="A_neuq_mt_negro.branchRates"/>
    </rateStatistic>
    <rateCovarianceStatistic id="A_neuq_mt_negro.covariance" name="A_neuq_mt_negro.covariance">
        <treeModel idref="A_neuq_mt_negro.treeModel"/>
        <discretizedBranchRates idref="A_neuq_mt_negro.branchRates"/>
    </rateCovarianceStatistic>

    <!-- The uncorrelated relaxed clock (Drummond, Ho, Phillips & Rambaut, 2006) -->
    <discretizedBranchRates id="A_neuq_nu_ANT_Negro.branchRates">
        <treeModel idref="A_neuq_nu_ANT_Negro.treeModel"/>
        <distribution>
            <logNormalDistributionModel meanInRealSpace="true">
                <mean>
```

```
                    <parameter id="A_neuq_nu_ANT_Negro.ucld.mean" value="0.6" lower="0.0" upper=
                </mean>
                <stdev>
                    <parameter id="A_neuq_nu_ANT_Negro.ucld.stdev" value="0.3333333333333333" lo
                </stdev>
            </logNormalDistributionModel>
        </distribution>
        <rateCategories>
            <parameter id="A_neuq_nu_ANT_Negro.branchRates.categories" dimension="250"/>
        </rateCategories>
    </discretizedBranchRates>
    <rateStatistic id="A_neuq_nu_ANT_Negro.meanRate" name="A_neuq_nu_ANT_Negro.meanRate" mode="m
        <treeModel idref="A_neuq_nu_ANT_Negro.treeModel"/>
        <discretizedBranchRates idref="A_neuq_nu_ANT_Negro.branchRates"/>
    </rateStatistic>
    <rateStatistic id="A_neuq_nu_ANT_Negro.coefficientOfVariation" name="A_neuq_nu_ANT_Negro.coe
        <treeModel idref="A_neuq_nu_ANT_Negro.treeModel"/>
        <discretizedBranchRates idref="A_neuq_nu_ANT_Negro.branchRates"/>
    </rateStatistic>
    <rateCovarianceStatistic id="A_neuq_nu_ANT_Negro.covariance" name="A_neuq_nu_ANT_Negro.covar
        <treeModel idref="A_neuq_nu_ANT_Negro.treeModel"/>
        <discretizedBranchRates idref="A_neuq_nu_ANT_Negro.branchRates"/>
    </rateCovarianceStatistic>

    <!-- The uncorrelated relaxed clock (Drummond, Ho, Phillips & Rambaut, 2006) -->
    <discretizedBranchRates id="A_neuq_nu_EF1Exon_Negro.branchRates">
        <treeModel idref="A_neuq_nu_EF1Exon_Negro.treeModel"/>
        <distribution>
            <logNormalDistributionModel meanInRealSpace="true">
                <mean>
                    <parameter id="A_neuq_nu_EF1Exon_Negro.ucld.mean" value="0.6" lower="0.0" up
                </mean>
                <stdev>
                    <parameter id="A_neuq_nu_EF1Exon_Negro.ucld.stdev" value="0.3333333333333333
                </stdev>
            </logNormalDistributionModel>
        </distribution>
        <rateCategories>
            <parameter id="A_neuq_nu_EF1Exon_Negro.branchRates.categories" dimension="294"/>
        </rateCategories>
    </discretizedBranchRates>
    <rateStatistic id="A_neuq_nu_EF1Exon_Negro.meanRate" name="A_neuq_nu_EF1Exon_Negro.meanRate"
        <treeModel idref="A_neuq_nu_EF1Exon_Negro.treeModel"/>
        <discretizedBranchRates idref="A_neuq_nu_EF1Exon_Negro.branchRates"/>
    </rateStatistic>
    <rateStatistic id="A_neuq_nu_EF1Exon_Negro.coefficientOfVariation" name="A_neuq_nu_EF1Exon_N
        <treeModel idref="A_neuq_nu_EF1Exon_Negro.treeModel"/>
        <discretizedBranchRates idref="A_neuq_nu_EF1Exon_Negro.branchRates"/>
    </rateStatistic>
    <rateCovarianceStatistic id="A_neuq_nu_EF1Exon_Negro.covariance" name="A_neuq_nu_EF1Exon_Neg
        <treeModel idref="A_neuq_nu_EF1Exon_Negro.treeModel"/>
        <discretizedBranchRates idref="A_neuq_nu_EF1Exon_Negro.branchRates"/>
    </rateCovarianceStatistic>

    <!-- The uncorrelated relaxed clock (Drummond, Ho, Phillips & Rambaut, 2006) -->
    <discretizedBranchRates id="A_neuq_nu_EF1Intron_Negro.branchRates">
        <treeModel idref="A_neuq_nu_EF1Intron_Negro.treeModel"/>
```

```
        <distribution>
            <logNormalDistributionModel meanInRealSpace="true">
                <mean>
                    <parameter id="A_neuq_nu_EF1Intron_Negro.ucld.mean" value="1.0" lower="0.0"
                </mean>
                <stdev>
                    <parameter id="A_neuq_nu_EF1Intron_Negro.ucld.stdev" value="0.3333333333333
                </stdev>
            </logNormalDistributionModel>
        </distribution>
        <rateCategories>
            <parameter id="A_neuq_nu_EF1Intron_Negro.branchRates.categories" dimension="246"/>
        </rateCategories>
    </discretizedBranchRates>
    <rateStatistic id="A_neuq_nu_EF1Intron_Negro.meanRate" name="A_neuq_nu_EF1Intron_Negro.meanR
        <treeModel idref="A_neuq_nu_EF1Intron_Negro.treeModel"/>
        <discretizedBranchRates idref="A_neuq_nu_EF1Intron_Negro.branchRates"/>
    </rateStatistic>
    <rateStatistic id="A_neuq_nu_EF1Intron_Negro.coefficientOfVariation" name="A_neuq_nu_EF1Intr
        <treeModel idref="A_neuq_nu_EF1Intron_Negro.treeModel"/>
        <discretizedBranchRates idref="A_neuq_nu_EF1Intron_Negro.branchRates"/>
    </rateStatistic>
    <rateCovarianceStatistic id="A_neuq_nu_EF1Intron_Negro.covariance" name="A_neuq_nu_EF1Intron
        <treeModel idref="A_neuq_nu_EF1Intron_Negro.treeModel"/>
        <discretizedBranchRates idref="A_neuq_nu_EF1Intron_Negro.branchRates"/>
    </rateCovarianceStatistic>

    <!-- The HKY substitution model (Hasegawa, Kishino & Yano, 1985)            -->
    <HKYModel id="A_neuq_mt_negro.hky">
        <frequencies>
            <frequencyModel dataType="nucleotide">
                <frequencies>
                    <parameter id="A_neuq_mt_negro.frequencies" value="0.25 0.25 0.25 0.25"/>
                </frequencies>
            </frequencyModel>
        </frequencies>
        <kappa>
            <parameter id="A_neuq_mt_negro.kappa" value="2.0" lower="0.0" upper="Infinity"/>
        </kappa>
    </HKYModel>

    <!-- site model                                                             -->
    <siteModel id="A_neuq_mt_negro.siteModel">
        <substitutionModel>
            <HKYModel idref="A_neuq_mt_negro.hky"/>
        </substitutionModel>
        <gammaShape gammaCategories="4">
            <parameter id="A_neuq_mt_negro.alpha" value="0.5" lower="0.0" upper="1000.0"/>
        </gammaShape>
        <proportionInvariant>
            <parameter id="A_neuq_mt_negro.pInv" value="0.5" lower="0.0" upper="1.0"/>
        </proportionInvariant>
    </siteModel>

    <!-- The general time reversible (GTR) substitution model                   -->
    <gtrModel id="A_neuq_nu_ANT_Negro.gtr">
        <frequencies>
```

```
            <frequencyModel dataType="nucleotide">
                <frequencies>
                    <parameter id="A_neuq_nu_ANT_Negro.frequencies" value="0.25 0.25 0.25 0.25"/
                </frequencies>
            </frequencyModel>
        </frequencies>
        <rateAC>
            <parameter id="A_neuq_nu_ANT_Negro.ac" value="1.0" lower="0.0" upper="Infinity"/>
        </rateAC>
        <rateAG>
            <parameter id="A_neuq_nu_ANT_Negro.ag" value="1.0" lower="0.0" upper="Infinity"/>
        </rateAG>
        <rateAT>
            <parameter id="A_neuq_nu_ANT_Negro.at" value="1.0" lower="0.0" upper="Infinity"/>
        </rateAT>
        <rateCG>
            <parameter id="A_neuq_nu_ANT_Negro.cg" value="1.0" lower="0.0" upper="Infinity"/>
        </rateCG>
        <rateGT>
            <parameter id="A_neuq_nu_ANT_Negro.gt" value="1.0" lower="0.0" upper="Infinity"/>
        </rateGT>
    </gtrModel>

    <!-- site model                                                                    -->
    <siteModel id="A_neuq_nu_ANT_Negro.siteModel">
        <substitutionModel>
            <gtrModel idref="A_neuq_nu_ANT_Negro.gtr"/>
        </substitutionModel>
        <proportionInvariant>
            <parameter id="A_neuq_nu_ANT_Negro.pInv" value="0.5" lower="0.0" upper="1.0"/>
        </proportionInvariant>
    </siteModel>

    <!-- The general time reversible (GTR) substitution model                          -->
    <gtrModel id="A_neuq_nu_EF1Exon_Negro.gtr">
        <frequencies>
            <frequencyModel dataType="nucleotide">
                <frequencies>
                    <parameter id="A_neuq_nu_EF1Exon_Negro.frequencies" value="0.25 0.25 0.25 0.
                </frequencies>
            </frequencyModel>
        </frequencies>
        <rateAC>
            <parameter id="A_neuq_nu_EF1Exon_Negro.ac" value="1.0" lower="0.0" upper="Infinity"/
        </rateAC>
        <rateAG>
            <parameter id="A_neuq_nu_EF1Exon_Negro.ag" value="1.0" lower="0.0" upper="Infinity"/
        </rateAG>
        <rateAT>
            <parameter id="A_neuq_nu_EF1Exon_Negro.at" value="1.0" lower="0.0" upper="Infinity"/
        </rateAT>
        <rateCG>
            <parameter id="A_neuq_nu_EF1Exon_Negro.cg" value="1.0" lower="0.0" upper="Infinity"/
        </rateCG>
        <rateGT>
            <parameter id="A_neuq_nu_EF1Exon_Negro.gt" value="1.0" lower="0.0" upper="Infinity"/
        </rateGT>
```

```
    </gtrModel>

    <!-- site model                                                                    -->
    <siteModel id="A_neuq_nu_EF1Exon_Negro.siteModel">
        <substitutionModel>
            <gtrModel idref="A_neuq_nu_EF1Exon_Negro.gtr"/>
        </substitutionModel>
        <proportionInvariant>
            <parameter id="A_neuq_nu_EF1Exon_Negro.pInv" value="0.5" lower="0.0" upper="1.0"/>
        </proportionInvariant>
    </siteModel>

    <!-- The HKY substitution model (Hasegawa, Kishino & Yano, 1985)             -->
    <HKYModel id="A_neuq_nu_EF1Intron_Negro.hky">
        <frequencies>
            <frequencyModel dataType="nucleotide">
                <frequencies>
                    <parameter id="A_neuq_nu_EF1Intron_Negro.frequencies" value="0.25 0.25 0.25
                </frequencies>
            </frequencyModel>
        </frequencies>
        <kappa>
            <parameter id="A_neuq_nu_EF1Intron_Negro.kappa" value="2.0" lower="0.0" upper="Infin
        </kappa>
    </HKYModel>

    <!-- site model                                                                    -->
    <siteModel id="A_neuq_nu_EF1Intron_Negro.siteModel">
        <substitutionModel>
            <HKYModel idref="A_neuq_nu_EF1Intron_Negro.hky"/>
        </substitutionModel>
        <gammaShape gammaCategories="4">
            <parameter id="A_neuq_nu_EF1Intron_Negro.alpha" value="0.5" lower="0.0" upper="1000.
        </gammaShape>
        <proportionInvariant>
            <parameter id="A_neuq_nu_EF1Intron_Negro.pInv" value="0.5" lower="0.0" upper="1.0"/>
        </proportionInvariant>
    </siteModel>
    <treeLikelihood id="A_neuq_mt_negro.treeLikelihood" useAmbiguities="false">
        <patterns idref="A_neuq_mt_negro.patterns"/>
        <treeModel idref="A_neuq_mt_negro.treeModel"/>
        <siteModel idref="A_neuq_mt_negro.siteModel"/>
        <discretizedBranchRates idref="A_neuq_mt_negro.branchRates"/>
    </treeLikelihood>
    <treeLikelihood id="A_neuq_nu_ANT_Negro.treeLikelihood" useAmbiguities="false">
        <patterns idref="A_neuq_nu_ANT_Negro.patterns"/>
        <treeModel idref="A_neuq_nu_ANT_Negro.treeModel"/>
        <siteModel idref="A_neuq_nu_ANT_Negro.siteModel"/>
        <discretizedBranchRates idref="A_neuq_nu_ANT_Negro.branchRates"/>
    </treeLikelihood>
    <treeLikelihood id="A_neuq_nu_EF1Exon_Negro.treeLikelihood" useAmbiguities="false">
        <patterns idref="A_neuq_nu_EF1Exon_Negro.patterns"/>
        <treeModel idref="A_neuq_nu_EF1Exon_Negro.treeModel"/>
        <siteModel idref="A_neuq_nu_EF1Exon_Negro.siteModel"/>
        <discretizedBranchRates idref="A_neuq_nu_EF1Exon_Negro.branchRates"/>
    </treeLikelihood>
    <treeLikelihood id="A_neuq_nu_EF1Intron_Negro.treeLikelihood" useAmbiguities="false">
```

```
        <patterns idref="A_neuq_nu_EF1Intron_Negro.patterns"/>
        <treeModel idref="A_neuq_nu_EF1Intron_Negro.treeModel"/>
        <siteModel idref="A_neuq_nu_EF1Intron_Negro.siteModel"/>
        <discretizedBranchRates idref="A_neuq_nu_EF1Intron_Negro.branchRates"/>
    </treeLikelihood>

    <!-- Define operators                                                   -->
    <operators id="operators">
        <scaleOperator scaleFactor="0.75" weight="2">
            <parameter idref="A_neuq_mt_negro.kappa"/>
        </scaleOperator>
        <deltaExchange delta="0.01" weight="0.1">
            <parameter idref="A_neuq_mt_negro.frequencies"/>
        </deltaExchange>
        <scaleOperator scaleFactor="0.75" weight="0.1">
            <parameter idref="A_neuq_mt_negro.alpha"/>
        </scaleOperator>
        <scaleOperator scaleFactor="0.75" weight="0.1">
            <parameter idref="A_neuq_mt_negro.pInv"/>
        </scaleOperator>
        <scaleOperator scaleFactor="0.75" weight="0.1">
            <parameter idref="A_neuq_nu_ANT_Negro.ac"/>
        </scaleOperator>
        <scaleOperator scaleFactor="0.75" weight="0.1">
            <parameter idref="A_neuq_nu_ANT_Negro.ag"/>
        </scaleOperator>
        <scaleOperator scaleFactor="0.75" weight="0.1">
            <parameter idref="A_neuq_nu_ANT_Negro.at"/>
        </scaleOperator>
        <scaleOperator scaleFactor="0.75" weight="0.1">
            <parameter idref="A_neuq_nu_ANT_Negro.cg"/>
        </scaleOperator>
        <scaleOperator scaleFactor="0.75" weight="0.1">
            <parameter idref="A_neuq_nu_ANT_Negro.gt"/>
        </scaleOperator>
        <deltaExchange delta="0.01" weight="0.1">
            <parameter idref="A_neuq_nu_ANT_Negro.frequencies"/>
        </deltaExchange>
        <scaleOperator scaleFactor="0.75" weight="0.1">
            <parameter idref="A_neuq_nu_ANT_Negro.pInv"/>
        </scaleOperator>
        <scaleOperator scaleFactor="0.75" weight="0.1">
            <parameter idref="A_neuq_nu_EF1Exon_Negro.pInv"/>
        </scaleOperator>
        <scaleOperator scaleFactor="0.75" weight="0.1">
            <parameter idref="A_neuq_nu_EF1Intron_Negro.kappa"/>
        </scaleOperator>
        <deltaExchange delta="0.01" weight="0.1">
            <parameter idref="A_neuq_nu_EF1Intron_Negro.frequencies"/>
        </deltaExchange>
        <scaleOperator scaleFactor="0.75" weight="0.1">
            <parameter idref="A_neuq_nu_EF1Intron_Negro.alpha"/>
        </scaleOperator>
        <scaleOperator scaleFactor="0.75" weight="0.1">
            <parameter idref="A_neuq_nu_EF1Intron_Negro.pInv"/>
        </scaleOperator>
        <scaleOperator scaleFactor="0.75" weight="3">
```

```
            <parameter idref="A_neuq_mt_negro.ucld.stdev"/>
        </scaleOperator>
        <scaleOperator scaleFactor="0.75" weight="3">
            <parameter idref="A_neuq_nu_ANT_Negro.ucld.mean"/>
        </scaleOperator>
        <scaleOperator scaleFactor="0.75" weight="3">
            <parameter idref="A_neuq_nu_ANT_Negro.ucld.stdev"/>
        </scaleOperator>
        <scaleOperator scaleFactor="0.75" weight="3">
            <parameter idref="A_neuq_nu_EF1Exon_Negro.ucld.mean"/>
        </scaleOperator>
        <scaleOperator scaleFactor="0.75" weight="3">
            <parameter idref="A_neuq_nu_EF1Exon_Negro.ucld.stdev"/>
        </scaleOperator>
        <scaleOperator scaleFactor="0.75" weight="3">
            <parameter idref="A_neuq_nu_EF1Intron_Negro.ucld.mean"/>
        </scaleOperator>
        <scaleOperator scaleFactor="0.75" weight="3">
            <parameter idref="A_neuq_nu_EF1Intron_Negro.ucld.stdev"/>
        </scaleOperator>
        <upDownOperator scaleFactor="0.75" weight="30">
            <up>
                <parameter idref="A_neuq_nu_ANT_Negro.ucld.mean"/>
                <parameter idref="A_neuq_nu_EF1Exon_Negro.ucld.mean"/>
                <parameter idref="A_neuq_nu_EF1Intron_Negro.ucld.mean"/>
            </up>
            <down>
                <parameter idref="demographic.popSize"/>
                <parameter idref="A_neuq_mt_negro.treeModel.allInternalNodeHeights"/>
                <parameter idref="A_neuq_nu_ANT_Negro.treeModel.allInternalNodeHeights"/>
                <parameter idref="A_neuq_nu_EF1Exon_Negro.treeModel.allInternalNodeHeights"/>
                <parameter idref="A_neuq_nu_EF1Intron_Negro.treeModel.allInternalNodeHeights"/>
            </down>
        </upDownOperator>
        <subtreeSlide size="0.02" gaussian="true" weight="15">
            <treeModel idref="A_neuq_mt_negro.treeModel"/>
        </subtreeSlide>
        <narrowExchange weight="15">
            <treeModel idref="A_neuq_mt_negro.treeModel"/>
        </narrowExchange>
        <wideExchange weight="3">
            <treeModel idref="A_neuq_mt_negro.treeModel"/>
        </wideExchange>
        <wilsonBalding weight="3">
            <treeModel idref="A_neuq_mt_negro.treeModel"/>
        </wilsonBalding>
        <scaleOperator scaleFactor="0.75" weight="3">
            <parameter idref="A_neuq_mt_negro.treeModel.rootHeight"/>
        </scaleOperator>
        <uniformOperator weight="30">
            <parameter idref="A_neuq_mt_negro.treeModel.internalNodeHeights"/>
        </uniformOperator>
        <subtreeSlide size="4.0E-4" gaussian="true" weight="15">
            <treeModel idref="A_neuq_nu_ANT_Negro.treeModel"/>
        </subtreeSlide>
        <narrowExchange weight="15">
            <treeModel idref="A_neuq_nu_ANT_Negro.treeModel"/>
```

```
        </narrowExchange>
        <wideExchange weight="3">
            <treeModel idref="A_neuq_nu_ANT_Negro.treeModel"/>
        </wideExchange>
        <wilsonBalding weight="3">
            <treeModel idref="A_neuq_nu_ANT_Negro.treeModel"/>
        </wilsonBalding>
        <scaleOperator scaleFactor="0.75" weight="3">
            <parameter idref="A_neuq_nu_ANT_Negro.treeModel.rootHeight"/>
        </scaleOperator>
        <uniformOperator weight="30">
            <parameter idref="A_neuq_nu_ANT_Negro.treeModel.internalNodeHeights"/>
        </uniformOperator>
        <subtreeSlide size="3.0000000000000003E-4" gaussian="true" weight="15">
            <treeModel idref="A_neuq_nu_EF1Exon_Negro.treeModel"/>
        </subtreeSlide>
        <narrowExchange weight="15">
            <treeModel idref="A_neuq_nu_EF1Exon_Negro.treeModel"/>
        </narrowExchange>
        <wideExchange weight="3">
            <treeModel idref="A_neuq_nu_EF1Exon_Negro.treeModel"/>
        </wideExchange>
        <wilsonBalding weight="3">
            <treeModel idref="A_neuq_nu_EF1Exon_Negro.treeModel"/>
        </wilsonBalding>
        <scaleOperator scaleFactor="0.75" weight="3">
            <parameter idref="A_neuq_nu_EF1Exon_Negro.treeModel.rootHeight"/>
        </scaleOperator>
        <uniformOperator weight="30">
            <parameter idref="A_neuq_nu_EF1Exon_Negro.treeModel.internalNodeHeights"/>
        </uniformOperator>
        <subtreeSlide size="0.0012000000000000001" gaussian="true" weight="15">
            <treeModel idref="A_neuq_nu_EF1Intron_Negro.treeModel"/>
        </subtreeSlide>
        <narrowExchange weight="15">
            <treeModel idref="A_neuq_nu_EF1Intron_Negro.treeModel"/>
        </narrowExchange>
        <wideExchange weight="3">
            <treeModel idref="A_neuq_nu_EF1Intron_Negro.treeModel"/>
        </wideExchange>
        <wilsonBalding weight="3">
            <treeModel idref="A_neuq_nu_EF1Intron_Negro.treeModel"/>
        </wilsonBalding>
        <scaleOperator scaleFactor="0.75" weight="3">
            <parameter idref="A_neuq_nu_EF1Intron_Negro.treeModel.rootHeight"/>
        </scaleOperator>
        <uniformOperator weight="30">
            <parameter idref="A_neuq_nu_EF1Intron_Negro.treeModel.internalNodeHeights"/>
        </uniformOperator>
        <scaleOperator scaleFactor="0.9" weight="3">
            <parameter idref="demographic.populationMean"/>
        </scaleOperator>
        <sampleNonActiveOperator weight="50">
            <distribution>
                <parameter idref="demographic.populationMeanDist"/>
            </distribution>
            <data>
```

```
            <parameter idref="demographic.popSize"/>
        </data>
        <indicators>
            <parameter idref="demographic.indicators"/>
        </indicators>
    </sampleNonActiveOperator>
    <bitFlipOperator weight="120">
        <parameter idref="demographic.indicators"/>
    </bitFlipOperator>
    <scaleOperator scaleFactor="0.5" weight="75">
        <parameter idref="demographic.popSize"/>
        <indicators pickoneprob="1.0">
            <parameter idref="demographic.indicators"/>
        </indicators>
    </scaleOperator>
    <upDownOperator scaleFactor="0.75" weight="15">
        <up>
        </up>
        <down>
            <parameter idref="A_neuq_mt_negro.treeModel.allInternalNodeHeights"/>
        </down>
    </upDownOperator>
    <swapOperator size="1" weight="10" autoOptimize="false">
        <parameter idref="A_neuq_mt_negro.branchRates.categories"/>
    </swapOperator>
    <randomWalkIntegerOperator windowSize="1" weight="10">
        <parameter idref="A_neuq_mt_negro.branchRates.categories"/>
    </randomWalkIntegerOperator>
    <uniformIntegerOperator weight="10">
        <parameter idref="A_neuq_mt_negro.branchRates.categories"/>
    </uniformIntegerOperator>
    <upDownOperator scaleFactor="0.75" weight="15">
        <up>
            <parameter idref="A_neuq_nu_ANT_Negro.ucld.mean"/>
        </up>
        <down>
            <parameter idref="A_neuq_nu_ANT_Negro.treeModel.allInternalNodeHeights"/>
        </down>
    </upDownOperator>
    <swapOperator size="1" weight="10" autoOptimize="false">
        <parameter idref="A_neuq_nu_ANT_Negro.branchRates.categories"/>
    </swapOperator>
    <randomWalkIntegerOperator windowSize="1" weight="10">
        <parameter idref="A_neuq_nu_ANT_Negro.branchRates.categories"/>
    </randomWalkIntegerOperator>
    <uniformIntegerOperator weight="10">
        <parameter idref="A_neuq_nu_ANT_Negro.branchRates.categories"/>
    </uniformIntegerOperator>
    <upDownOperator scaleFactor="0.75" weight="15">
        <up>
            <parameter idref="A_neuq_nu_EF1Exon_Negro.ucld.mean"/>
        </up>
        <down>
            <parameter idref="A_neuq_nu_EF1Exon_Negro.treeModel.allInternalNodeHeights"/>
        </down>
    </upDownOperator>
    <swapOperator size="1" weight="10" autoOptimize="false">
```

```
            <parameter idref="A_neuq_nu_EF1Exon_Negro.branchRates.categories"/>
        </swapOperator>
        <randomWalkIntegerOperator windowSize="1" weight="10">
            <parameter idref="A_neuq_nu_EF1Exon_Negro.branchRates.categories"/>
        </randomWalkIntegerOperator>
        <uniformIntegerOperator weight="10">
            <parameter idref="A_neuq_nu_EF1Exon_Negro.branchRates.categories"/>
        </uniformIntegerOperator>
        <upDownOperator scaleFactor="0.75" weight="15">
            <up>
                <parameter idref="A_neuq_nu_EF1Intron_Negro.ucld.mean"/>
            </up>
            <down>
                <parameter idref="A_neuq_nu_EF1Intron_Negro.treeModel.allInternalNodeHeights"/>
            </down>
        </upDownOperator>
        <swapOperator size="1" weight="10" autoOptimize="false">
            <parameter idref="A_neuq_nu_EF1Intron_Negro.branchRates.categories"/>
        </swapOperator>
        <randomWalkIntegerOperator windowSize="1" weight="10">
            <parameter idref="A_neuq_nu_EF1Intron_Negro.branchRates.categories"/>
        </randomWalkIntegerOperator>
        <uniformIntegerOperator weight="10">
            <parameter idref="A_neuq_nu_EF1Intron_Negro.branchRates.categories"/>
        </uniformIntegerOperator>
    </operators>

    <!-- Define MCMC                                                          -->
    <mcmc id="mcmc" chainLength="50000000" autoOptimize="true">
        <posterior id="posterior">
            <prior id="prior">
                <logNormalPrior mean="1.0" stdev="1.25" offset="0.0" meanInRealSpace="false">
                    <parameter idref="A_neuq_mt_negro.kappa"/>
                </logNormalPrior>
                <gammaPrior shape="0.05" scale="10.0" offset="0.0">
                    <parameter idref="A_neuq_nu_ANT_Negro.ac"/>
                </gammaPrior>
                <gammaPrior shape="0.05" scale="20.0" offset="0.0">
                    <parameter idref="A_neuq_nu_ANT_Negro.ag"/>
                </gammaPrior>
                <gammaPrior shape="0.05" scale="10.0" offset="0.0">
                    <parameter idref="A_neuq_nu_ANT_Negro.at"/>
                </gammaPrior>
                <gammaPrior shape="0.05" scale="10.0" offset="0.0">
                    <parameter idref="A_neuq_nu_ANT_Negro.cg"/>
                </gammaPrior>
                <gammaPrior shape="0.05" scale="10.0" offset="0.0">
                    <parameter idref="A_neuq_nu_ANT_Negro.gt"/>
                </gammaPrior>
                <gammaPrior shape="0.05" scale="10.0" offset="0.0">
                    <parameter idref="A_neuq_nu_EF1Exon_Negro.ac"/>
                </gammaPrior>
                <gammaPrior shape="0.05" scale="20.0" offset="0.0">
                    <parameter idref="A_neuq_nu_EF1Exon_Negro.ag"/>
                </gammaPrior>
                <gammaPrior shape="0.05" scale="10.0" offset="0.0">
                    <parameter idref="A_neuq_nu_EF1Exon_Negro.at"/>
```

```
            </gammaPrior>
            <gammaPrior shape="0.05" scale="10.0" offset="0.0">
                <parameter idref="A_neuq_nu_EF1Exon_Negro.cg"/>
            </gammaPrior>
            <gammaPrior shape="0.05" scale="10.0" offset="0.0">
                <parameter idref="A_neuq_nu_EF1Exon_Negro.gt"/>
            </gammaPrior>
            <logNormalPrior mean="1.0" stdev="1.25" offset="0.0" meanInRealSpace="false">
                <parameter idref="A_neuq_nu_EF1Intron_Negro.kappa"/>
            </logNormalPrior>
            <exponentialPrior mean="0.3333333333333333" offset="0.0">
                <parameter idref="A_neuq_mt_negro.ucld.stdev"/>
            </exponentialPrior>
            <exponentialPrior mean="0.3333333333333333" offset="0.0">
                <parameter idref="A_neuq_nu_ANT_Negro.ucld.stdev"/>
            </exponentialPrior>
            <logNormalPrior mean="0.0" stdev="1.0" offset="0.0" meanInRealSpace="false">
                <parameter idref="A_neuq_nu_ANT_Negro.ucld.mean"/>
            </logNormalPrior>
            <exponentialPrior mean="0.3333333333333333" offset="0.0">
                <parameter idref="A_neuq_nu_EF1Exon_Negro.ucld.stdev"/>
            </exponentialPrior>
            <logNormalPrior mean="0.0" stdev="1.0" offset="0.0" meanInRealSpace="false">
                <parameter idref="A_neuq_nu_EF1Exon_Negro.ucld.mean"/>
            </logNormalPrior>
            <exponentialPrior mean="0.3333333333333333" offset="0.0">
                <parameter idref="A_neuq_nu_EF1Intron_Negro.ucld.stdev"/>
            </exponentialPrior>
            <logNormalPrior mean="0.0" stdev="1.0" offset="0.0" meanInRealSpace="false">
                <parameter idref="A_neuq_nu_EF1Intron_Negro.ucld.mean"/>
            </logNormalPrior>
            <poissonPrior mean="0.6931471805599453" offset="0.0">
                <statistic idref="demographic.populationSizeChanges"/>
            </poissonPrior>
            <oneOnXPrior>
                <parameter idref="demographic.populationMean"/>
            </oneOnXPrior>
            <coalescentLikelihood idref="coalescent"/>
            <mixedDistributionLikelihood>
                <distribution0>
                    <exponentialDistributionModel idref="demographic.populationMeanDist"/>
                </distribution0>
                <distribution1>
                    <exponentialDistributionModel idref="demographic.populationMeanDist"/>
                </distribution1>
                <data>
                    <parameter idref="demographic.popSize"/>
                </data>
                <indicators>
                    <parameter idref="demographic.indicators"/>
                </indicators>
            </mixedDistributionLikelihood>
        </prior>
        <likelihood id="likelihood">
            <treeLikelihood idref="A_neuq_mt_negro.treeLikelihood"/>
            <treeLikelihood idref="A_neuq_nu_ANT_Negro.treeLikelihood"/>
            <treeLikelihood idref="A_neuq_nu_EF1Exon_Negro.treeLikelihood"/>
```

```
            <treeLikelihood idref="A_neuq_nu_EF1Intron_Negro.treeLikelihood"/>
        </likelihood>
    </posterior>
    <operators idref="operators"/>

    <!-- write log to screen                                                 -->
    <log id="screenLog" logEvery="2000">
        <column label="Posterior" dp="4" width="12">
            <posterior idref="posterior"/>
        </column>
        <column label="Prior" dp="4" width="12">
            <prior idref="prior"/>
        </column>
        <column label="Likelihood" dp="4" width="12">
            <likelihood idref="likelihood"/>
        </column>
        <column label="A_neuq_mt_negro.rootHeight" sf="6" width="12">
            <parameter idref="A_neuq_mt_negro.treeModel.rootHeight"/>
        </column>
        <column label="A_neuq_nu_ANT_Negro.rootHeight" sf="6" width="12">
            <parameter idref="A_neuq_nu_ANT_Negro.treeModel.rootHeight"/>
        </column>
        <column label="A_neuq_nu_EF1Exon_Negro.rootHeight" sf="6" width="12">
            <parameter idref="A_neuq_nu_EF1Exon_Negro.treeModel.rootHeight"/>
        </column>
        <column label="A_neuq_nu_EF1Intron_Negro.rootHeight" sf="6" width="12">
            <parameter idref="A_neuq_nu_EF1Intron_Negro.treeModel.rootHeight"/>
        </column>
        <column label="A_neuq_mt_negro.ucld.mean" sf="6" width="12">
            <parameter idref="A_neuq_mt_negro.ucld.mean"/>
        </column>
        <column label="A_neuq_nu_ANT_Negro.ucld.mean" sf="6" width="12">
            <parameter idref="A_neuq_nu_ANT_Negro.ucld.mean"/>
        </column>
        <column label="A_neuq_nu_EF1Exon_Negro.ucld.mean" sf="6" width="12">
            <parameter idref="A_neuq_nu_EF1Exon_Negro.ucld.mean"/>
        </column>
        <column label="A_neuq_nu_EF1Intron_Negro.ucld.mean" sf="6" width="12">
            <parameter idref="A_neuq_nu_EF1Intron_Negro.ucld.mean"/>
        </column>
    </log>

    <!-- write log to file                                                   -->
    <log id="fileLog" logEvery="2000" fileName="A_neuq_All_Run1_negro.log" overwrite="false"
        <posterior idref="posterior"/>
        <prior idref="prior"/>
        <likelihood idref="likelihood"/>
        <parameter idref="A_neuq_mt_negro.treeModel.rootHeight"/>
        <parameter idref="A_neuq_nu_ANT_Negro.treeModel.rootHeight"/>
        <parameter idref="A_neuq_nu_EF1Exon_Negro.treeModel.rootHeight"/>
        <parameter idref="A_neuq_nu_EF1Intron_Negro.treeModel.rootHeight"/>
        <sumStatistic idref="demographic.populationSizeChanges"/>
        <parameter idref="demographic.populationMean"/>
        <parameter idref="demographic.popSize"/>
        <parameter idref="demographic.indicators"/>
        <parameter idref="A_neuq_mt_negro.kappa"/>
        <parameter idref="A_neuq_mt_negro.frequencies"/>
```

```
            <parameter idref="A_neuq_mt_negro.alpha"/>
            <parameter idref="A_neuq_mt_negro.pInv"/>
            <parameter idref="A_neuq_nu_ANT_Negro.ac"/>
            <parameter idref="A_neuq_nu_ANT_Negro.ag"/>
            <parameter idref="A_neuq_nu_ANT_Negro.at"/>
            <parameter idref="A_neuq_nu_ANT_Negro.cg"/>
            <parameter idref="A_neuq_nu_ANT_Negro.gt"/>
            <parameter idref="A_neuq_nu_ANT_Negro.frequencies"/>
            <parameter idref="A_neuq_nu_ANT_Negro.pInv"/>
            <parameter idref="A_neuq_nu_EF1Exon_Negro.ac"/>
            <parameter idref="A_neuq_nu_EF1Exon_Negro.ag"/>
            <parameter idref="A_neuq_nu_EF1Exon_Negro.at"/>
            <parameter idref="A_neuq_nu_EF1Exon_Negro.cg"/>
            <parameter idref="A_neuq_nu_EF1Exon_Negro.gt"/>
            <parameter idref="A_neuq_nu_EF1Exon_Negro.pInv"/>
            <parameter idref="A_neuq_nu_EF1Intron_Negro.kappa"/>
            <parameter idref="A_neuq_nu_EF1Intron_Negro.frequencies"/>
            <parameter idref="A_neuq_nu_EF1Intron_Negro.alpha"/>
            <parameter idref="A_neuq_nu_EF1Intron_Negro.pInv"/>
            <parameter idref="A_neuq_mt_negro.ucld.mean"/>
            <parameter idref="A_neuq_mt_negro.ucld.stdev"/>
            <parameter idref="A_neuq_nu_ANT_Negro.ucld.mean"/>
            <parameter idref="A_neuq_nu_ANT_Negro.ucld.stdev"/>
            <parameter idref="A_neuq_nu_EF1Exon_Negro.ucld.mean"/>
            <parameter idref="A_neuq_nu_EF1Exon_Negro.ucld.stdev"/>
            <parameter idref="A_neuq_nu_EF1Intron_Negro.ucld.mean"/>
            <parameter idref="A_neuq_nu_EF1Intron_Negro.ucld.stdev"/>
            <rateStatistic idref="A_neuq_mt_negro.meanRate"/>
            <rateStatistic idref="A_neuq_mt_negro.coefficientOfVariation"/>
            <rateCovarianceStatistic idref="A_neuq_mt_negro.covariance"/>
            <rateStatistic idref="A_neuq_nu_ANT_Negro.meanRate"/>
            <rateStatistic idref="A_neuq_nu_ANT_Negro.coefficientOfVariation"/>
            <rateCovarianceStatistic idref="A_neuq_nu_ANT_Negro.covariance"/>
            <rateStatistic idref="A_neuq_nu_EF1Exon_Negro.meanRate"/>
            <rateStatistic idref="A_neuq_nu_EF1Exon_Negro.coefficientOfVariation"/>
            <rateCovarianceStatistic idref="A_neuq_nu_EF1Exon_Negro.covariance"/>
            <rateStatistic idref="A_neuq_nu_EF1Intron_Negro.meanRate"/>
            <rateStatistic idref="A_neuq_nu_EF1Intron_Negro.coefficientOfVariation"/>
            <rateCovarianceStatistic idref="A_neuq_nu_EF1Intron_Negro.covariance"/>
            <treeLikelihood idref="A_neuq_mt_negro.treeLikelihood"/>
            <treeLikelihood idref="A_neuq_nu_ANT_Negro.treeLikelihood"/>
            <treeLikelihood idref="A_neuq_nu_EF1Exon_Negro.treeLikelihood"/>
            <treeLikelihood idref="A_neuq_nu_EF1Intron_Negro.treeLikelihood"/>
            <coalescentLikelihood idref="coalescent"/>
        </log>

        <!-- write tree log to file                                                -->
        <logTree id="A_neuq_mt_negro.treeFileLog" logEvery="2000" nexusFormat="true" fileName="A
            <treeModel idref="A_neuq_mt_negro.treeModel"/>
            <discretizedBranchRates idref="A_neuq_mt_negro.branchRates"/>
            <posterior idref="posterior"/>
        </logTree>
        <logTree id="A_neuq_nu_ANT_Negro.treeFileLog" logEvery="2000" nexusFormat="true" fileNam
            <treeModel idref="A_neuq_nu_ANT_Negro.treeModel"/>
            <discretizedBranchRates idref="A_neuq_nu_ANT_Negro.branchRates"/>
            <posterior idref="posterior"/>
        </logTree>
```

```
        <logTree id="A_neuq_nu_EF1Exon_Negro.treeFileLog" logEvery="2000" nexusFormat="true" fil
            <treeModel idref="A_neuq_nu_EF1Exon_Negro.treeModel"/>
            <discretizedBranchRates idref="A_neuq_nu_EF1Exon_Negro.branchRates"/>
            <posterior idref="posterior"/>
        </logTree>
        <logTree id="A_neuq_nu_EF1Intron_Negro.treeFileLog" logEvery="2000" nexusFormat="true" f
            <treeModel idref="A_neuq_nu_EF1Intron_Negro.treeModel"/>
            <discretizedBranchRates idref="A_neuq_nu_EF1Intron_Negro.branchRates"/>
            <posterior idref="posterior"/>
        </logTree>
    </mcmc>
    <report>
        <property name="timer">
            <mcmc idref="mcmc"/>
        </property>
    </report>
    <VDAnalysis id="demographic.analysis" burnIn="0.2" useMidpoints="true">
        <logFileName>
            A_neuq_All_Run1_negro.log
        </logFileName>
        <treeFileNames>
            <treeOfLoci>
                A_neuq_All_Run1_negro.A_neuq_mt_negro.trees
            </treeOfLoci>
            <treeOfLoci>
                A_neuq_All_Run1_negro.A_neuq_nu_ANT_Negro.trees
            </treeOfLoci>
            <treeOfLoci>
                A_neuq_All_Run1_negro.A_neuq_nu_EF1Exon_Negro.trees
            </treeOfLoci>
            <treeOfLoci>
                A_neuq_All_Run1_negro.A_neuq_nu_EF1Intron_Negro.trees
            </treeOfLoci>
        </treeFileNames>
        <populationModelType>
            linear
        </populationModelType>
        <populationFirstColumn>
            demographic.popSize1
        </populationFirstColumn>
        <indicatorsFirstColumn>
            demographic.indicators1
        </indicatorsFirstColumn>
    </VDAnalysis>
    <CSVexport fileName="A_neuq_All_Run1_negro.csv" separator=",">
        <columns>
            <VDAnalysis idref="demographic.analysis"/>
        </columns>
    </CSVexport>
</beast>
```
