# Supplementary material for: Conflicting Evolutionary Patterns Due to Mitochondrial Introgression and Multilocus Phylogeography of the Patagonian Freshwater Crab Aegla neuquensis
Source: PLoS One. 2012 Jun 7;7(6):e37105. doi: 10.1371/journal.pone.0037105 (PMC3369872; doi:10.1371/journal.pone.0037105)
Supplement: Data File S2 — Beast input file (.xml) for Extended Bayesian Skyline Plot analysis of the Chico-Chubut River system. (PDF) [file pone.0037105.s003.pdf]

```xml
<?xml version="1.0" standalone="yes"?>

<!-- Generated by BEAUTi v1.6.1                                              -->
<!--       by Alexei J. Drummond and Andrew Rambaut                          -->
<!--       Department of Computer Science, University of Auckland and        -->
<!--       Institute of Evolutionary Biology, University of Edinburgh        -->
<!--       http://beast.bio.ed.ac.uk/                                        -->
<beast>

    <!-- The list of taxa analyse (can also include dates/ages).                 -->
    <!-- ntax=211                                                                -->
    <taxa id="taxa">
    ****DATA section removed to reduce file size*****

    </alignment>

    <!-- The unique patterns from 1 to end                                       -->
    <!-- npatterns=48                                                            -->
    <patterns id="A_neuq_mt_chubut.patterns" from="1">
        <alignment idref="alignment1"/>
    </patterns>

    <!-- The unique patterns from 1 to end                                       -->
    <!-- npatterns=6                                                             -->
    <patterns id="A_neuq_nu_ANT_Chubut.patterns" from="1">
        <alignment idref="alignment2"/>
    </patterns>

    <!-- The unique patterns from 1 to end                                       -->
    <!-- npatterns=4                                                             -->
    <patterns id="A_neuq_nu_EF1Exon_Chubut.patterns" from="1">
        <alignment idref="alignment3"/>
    </patterns>

    <!-- The unique patterns from 1 to end                                       -->
    <!-- npatterns=7                                                             -->
    <patterns id="A_neuq_nu_EF1Intron_Chubut.patterns" from="1">
        <alignment idref="alignment4"/>
    </patterns>

    <!-- This is a simple constant population size coalescent model              -->
    <!-- that is used to generate an initial tree for the chain.                 -->
    <constantSize id="initialDemo" units="substitutions">
        <populationSize>
            <parameter id="initialDemo.popSize" value="100.0"/>
        </populationSize>
    </constantSize>

    <!-- Generate a random starting tree under the coalescent process            -->
    <coalescentTree id="A_neuq_mt_chubut.startingTree" rootHeight="0.017">
        <taxa idref="A_neuq_mt_chubut.taxa"/>
        <constantSize idref="initialDemo"/>
    </coalescentTree>

    <!-- Generate a random starting tree under the coalescent process            -->
    <coalescentTree id="A_neuq_nu_ANT_Chubut.startingTree" rootHeight="0.0012">
        <taxa idref="A_neuq_nu_ANT_Chubut.taxa"/>
```

```
        <constantSize idref="initialDemo"/>
    </coalescentTree>

    <!-- Generate a random starting tree under the coalescent process            -->
    <coalescentTree id="A_neuq_nu_EF1Exon_Chubut.startingTree" rootHeight="0.0">
        <taxa idref="A_neuq_nu_EF1Exon_Chubut.taxa"/>
        <constantSize idref="initialDemo"/>
    </coalescentTree>

    <!-- Generate a random starting tree under the coalescent process            -->
    <coalescentTree id="A_neuq_nu_EF1Intron_Chubut.startingTree" rootHeight="0.0">
        <taxa idref="A_neuq_nu_EF1Intron_Chubut.taxa"/>
        <constantSize idref="initialDemo"/>
    </coalescentTree>

    <!-- Generate a tree model                                                   -->
    <treeModel id="A_neuq_mt_chubut.treeModel">
        <coalescentTree idref="A_neuq_mt_chubut.startingTree"/>
        <rootHeight>
            <parameter id="A_neuq_mt_chubut.treeModel.rootHeight"/>
        </rootHeight>
        <nodeHeights internalNodes="true">
            <parameter id="A_neuq_mt_chubut.treeModel.internalNodeHeights"/>
        </nodeHeights>
        <nodeHeights internalNodes="true" rootNode="true">
            <parameter id="A_neuq_mt_chubut.treeModel.allInternalNodeHeights"/>
        </nodeHeights>
    </treeModel>

    <!-- Generate a tree model                                                   -->
    <treeModel id="A_neuq_nu_ANT_Chubut.treeModel">
        <coalescentTree idref="A_neuq_nu_ANT_Chubut.startingTree"/>
        <rootHeight>
            <parameter id="A_neuq_nu_ANT_Chubut.treeModel.rootHeight"/>
        </rootHeight>
        <nodeHeights internalNodes="true">
            <parameter id="A_neuq_nu_ANT_Chubut.treeModel.internalNodeHeights"/>
        </nodeHeights>
        <nodeHeights internalNodes="true" rootNode="true">
            <parameter id="A_neuq_nu_ANT_Chubut.treeModel.allInternalNodeHeights"/>
        </nodeHeights>
    </treeModel>

    <!-- Generate a tree model                                                   -->
    <treeModel id="A_neuq_nu_EF1Exon_Chubut.treeModel">
        <coalescentTree idref="A_neuq_nu_EF1Exon_Chubut.startingTree"/>
        <rootHeight>
            <parameter id="A_neuq_nu_EF1Exon_Chubut.treeModel.rootHeight"/>
        </rootHeight>
        <nodeHeights internalNodes="true">
            <parameter id="A_neuq_nu_EF1Exon_Chubut.treeModel.internalNodeHeights"/>
        </nodeHeights>
        <nodeHeights internalNodes="true" rootNode="true">
            <parameter id="A_neuq_nu_EF1Exon_Chubut.treeModel.allInternalNodeHeights"/>
        </nodeHeights>
    </treeModel>
```

```
    <!-- Generate a tree model                                                   -->
    <treeModel id="A_neuq_nu_EF1Intron_Chubut.treeModel">
        <coalescentTree idref="A_neuq_nu_EF1Intron_Chubut.startingTree"/>
        <rootHeight>
            <parameter id="A_neuq_nu_EF1Intron_Chubut.treeModel.rootHeight"/>
        </rootHeight>
        <nodeHeights internalNodes="true">
            <parameter id="A_neuq_nu_EF1Intron_Chubut.treeModel.internalNodeHeights"/>
        </nodeHeights>
        <nodeHeights internalNodes="true" rootNode="true">
            <parameter id="A_neuq_nu_EF1Intron_Chubut.treeModel.allInternalNodeHeights"/>
        </nodeHeights>
    </treeModel>

    <!-- Generate a variableDemographic for extended Bayesian skyline process    -->
    <variableDemographic id="demographic" type="linear" useMidpoints="true">
        <populationSizes>

            <!-- popSize value = populationMean value                                    -->
            <parameter id="demographic.popSize" value="1.0"/>
        </populationSizes>
        <indicators>
            <parameter id="demographic.indicators" value="0.0"/>
        </indicators>
        <trees>
            <ptree ploidy="0.5">
                <treeModel idref="A_neuq_mt_chubut.treeModel"/>
            </ptree>
            <ptree ploidy="2.0">
                <treeModel idref="A_neuq_nu_ANT_Chubut.treeModel"/>
            </ptree>
            <ptree ploidy="2.0">
                <treeModel idref="A_neuq_nu_EF1Exon_Chubut.treeModel"/>
            </ptree>
            <ptree ploidy="2.0">
                <treeModel idref="A_neuq_nu_EF1Intron_Chubut.treeModel"/>
            </ptree>
        </trees>
    </variableDemographic>
    <coalescentLikelihood id="coalescent">
        <model>
            <variableDemographic idref="demographic"/>
        </model>

        <!-- Take population Tree from demographic                                   -->
    </coalescentLikelihood>
    <sumStatistic id="demographic.populationSizeChanges" elementwise="true">
        <parameter idref="demographic.indicators"/>
    </sumStatistic>
    <exponentialDistributionModel id="demographic.populationMeanDist">
        <mean>

            <!-- prefer populationMean value = 1                                         -->
            <parameter id="demographic.populationMean" value="1.0"/>
        </mean>
    </exponentialDistributionModel>
```

```
    <!-- The uncorrelated relaxed clock (Drummond, Ho, Phillips & Rambaut, 2006) -->
    <discretizedBranchRates id="A_neuq_mt_chubut.branchRates">
        <treeModel idref="A_neuq_mt_chubut.treeModel"/>
        <distribution>
            <logNormalDistributionModel meanInRealSpace="true">
                <mean>
                    <parameter id="A_neuq_mt_chubut.ucld.mean" value="0.118"/>
                </mean>
                <stdev>
                    <parameter id="A_neuq_mt_chubut.ucld.stdev" value="0.3333333333333333" lower
                </stdev>
            </logNormalDistributionModel>
        </distribution>
        <rateCategories>
            <parameter id="A_neuq_mt_chubut.branchRates.categories" dimension="206"/>
        </rateCategories>
    </discretizedBranchRates>
    <rateStatistic id="A_neuq_mt_chubut.meanRate" name="A_neuq_mt_chubut.meanRate" mode="mean" i
        <treeModel idref="A_neuq_mt_chubut.treeModel"/>
        <discretizedBranchRates idref="A_neuq_mt_chubut.branchRates"/>
    </rateStatistic>
    <rateStatistic id="A_neuq_mt_chubut.coefficientOfVariation" name="A_neuq_mt_chubut.coefficie
        <treeModel idref="A_neuq_mt_chubut.treeModel"/>
        <discretizedBranchRates idref="A_neuq_mt_chubut.branchRates"/>
    </rateStatistic>
    <rateCovarianceStatistic id="A_neuq_mt_chubut.covariance" name="A_neuq_mt_chubut.covariance"
        <treeModel idref="A_neuq_mt_chubut.treeModel"/>
        <discretizedBranchRates idref="A_neuq_mt_chubut.branchRates"/>
    </rateCovarianceStatistic>

    <!-- The uncorrelated relaxed clock (Drummond, Ho, Phillips & Rambaut, 2006) -->
    <discretizedBranchRates id="A_neuq_nu_ANT_Chubut.branchRates">
        <treeModel idref="A_neuq_nu_ANT_Chubut.treeModel"/>
        <distribution>
            <logNormalDistributionModel meanInRealSpace="true">
                <mean>
                    <parameter id="A_neuq_nu_ANT_Chubut.ucld.mean" value="0.1" lower="0.0" upper
                </mean>
                <stdev>
                    <parameter id="A_neuq_nu_ANT_Chubut.ucld.stdev" value="0.3333333333333333" l
                </stdev>
            </logNormalDistributionModel>
        </distribution>
        <rateCategories>
            <parameter id="A_neuq_nu_ANT_Chubut.branchRates.categories" dimension="98"/>
        </rateCategories>
    </discretizedBranchRates>
    <rateStatistic id="A_neuq_nu_ANT_Chubut.meanRate" name="A_neuq_nu_ANT_Chubut.meanRate" mode=
        <treeModel idref="A_neuq_nu_ANT_Chubut.treeModel"/>
        <discretizedBranchRates idref="A_neuq_nu_ANT_Chubut.branchRates"/>
    </rateStatistic>
    <rateStatistic id="A_neuq_nu_ANT_Chubut.coefficientOfVariation" name="A_neuq_nu_ANT_Chubut.c
        <treeModel idref="A_neuq_nu_ANT_Chubut.treeModel"/>
        <discretizedBranchRates idref="A_neuq_nu_ANT_Chubut.branchRates"/>
    </rateStatistic>
    <rateCovarianceStatistic id="A_neuq_nu_ANT_Chubut.covariance" name="A_neuq_nu_ANT_Chubut.cov
        <treeModel idref="A_neuq_nu_ANT_Chubut.treeModel"/>
```

```
        <discretizedBranchRates idref="A_neuq_nu_ANT_Chubut.branchRates"/>
    </rateCovarianceStatistic>

    <!-- The uncorrelated relaxed clock (Drummond, Ho, Phillips & Rambaut, 2006) -->
    <discretizedBranchRates id="A_neuq_nu_EF1Exon_Chubut.branchRates">
        <treeModel idref="A_neuq_nu_EF1Exon_Chubut.treeModel"/>
        <distribution>
            <logNormalDistributionModel meanInRealSpace="true">
                <mean>
                    <parameter id="A_neuq_nu_EF1Exon_Chubut.ucld.mean" value="0.1" lower="0.0" u
                </mean>
                <stdev>
                    <parameter id="A_neuq_nu_EF1Exon_Chubut.ucld.stdev" value="0.333333333333333
                </stdev>
            </logNormalDistributionModel>
        </distribution>
        <rateCategories>
            <parameter id="A_neuq_nu_EF1Exon_Chubut.branchRates.categories" dimension="98"/>
        </rateCategories>
    </discretizedBranchRates>
    <rateStatistic id="A_neuq_nu_EF1Exon_Chubut.meanRate" name="A_neuq_nu_EF1Exon_Chubut.meanRat
        <treeModel idref="A_neuq_nu_EF1Exon_Chubut.treeModel"/>
        <discretizedBranchRates idref="A_neuq_nu_EF1Exon_Chubut.branchRates"/>
    </rateStatistic>
    <rateStatistic id="A_neuq_nu_EF1Exon_Chubut.coefficientOfVariation" name="A_neuq_nu_EF1Exon_
        <treeModel idref="A_neuq_nu_EF1Exon_Chubut.treeModel"/>
        <discretizedBranchRates idref="A_neuq_nu_EF1Exon_Chubut.branchRates"/>
    </rateStatistic>
    <rateCovarianceStatistic id="A_neuq_nu_EF1Exon_Chubut.covariance" name="A_neuq_nu_EF1Exon_Ch
        <treeModel idref="A_neuq_nu_EF1Exon_Chubut.treeModel"/>
        <discretizedBranchRates idref="A_neuq_nu_EF1Exon_Chubut.branchRates"/>
    </rateCovarianceStatistic>

    <!-- The uncorrelated relaxed clock (Drummond, Ho, Phillips & Rambaut, 2006) -->
    <discretizedBranchRates id="A_neuq_nu_EF1Intron_Chubut.branchRates">
        <treeModel idref="A_neuq_nu_EF1Intron_Chubut.treeModel"/>
        <distribution>
            <logNormalDistributionModel meanInRealSpace="true">
                <mean>
                    <parameter id="A_neuq_nu_EF1Intron_Chubut.ucld.mean" value="0.1" lower="0.0"
                </mean>
                <stdev>
                    <parameter id="A_neuq_nu_EF1Intron_Chubut.ucld.stdev" value="0.3333333333333
                </stdev>
            </logNormalDistributionModel>
        </distribution>
        <rateCategories>
            <parameter id="A_neuq_nu_EF1Intron_Chubut.branchRates.categories" dimension="94"/>
        </rateCategories>
    </discretizedBranchRates>
    <rateStatistic id="A_neuq_nu_EF1Intron_Chubut.meanRate" name="A_neuq_nu_EF1Intron_Chubut.mea
        <treeModel idref="A_neuq_nu_EF1Intron_Chubut.treeModel"/>
        <discretizedBranchRates idref="A_neuq_nu_EF1Intron_Chubut.branchRates"/>
    </rateStatistic>
    <rateStatistic id="A_neuq_nu_EF1Intron_Chubut.coefficientOfVariation" name="A_neuq_nu_EF1Int
        <treeModel idref="A_neuq_nu_EF1Intron_Chubut.treeModel"/>
        <discretizedBranchRates idref="A_neuq_nu_EF1Intron_Chubut.branchRates"/>
```

```xml
	</rateStatistic>
	<rateCovarianceStatistic id="A_neuq_nu_EF1Intron_Chubut.covariance" name="A_neuq_nu_EF1Intro
		<treeModel idref="A_neuq_nu_EF1Intron_Chubut.treeModel"/>
		<discretizedBranchRates idref="A_neuq_nu_EF1Intron_Chubut.branchRates"/>
	</rateCovarianceStatistic>

	<!-- The HKY substitution model (Hasegawa, Kishino & Yano, 1985)           -->
	<HKYModel id="A_neuq_mt_chubut.hky">
		<frequencies>
			<frequencyModel dataType="nucleotide">
				<frequencies>
					<parameter id="A_neuq_mt_chubut.frequencies" value="0.25 0.25 0.25 0.25"/>
				</frequencies>
			</frequencyModel>
		</frequencies>
		<kappa>
			<parameter id="A_neuq_mt_chubut.kappa" value="2.0" lower="0.0" upper="Infinity"/>
		</kappa>
	</HKYModel>

	<!-- site model                                                              -->
	<siteModel id="A_neuq_mt_chubut.siteModel">
		<substitutionModel>
			<HKYModel idref="A_neuq_mt_chubut.hky"/>
		</substitutionModel>
		<gammaShape gammaCategories="4">
			<parameter id="A_neuq_mt_chubut.alpha" value="0.5" lower="0.0" upper="1000.0"/>
		</gammaShape>
		<proportionInvariant>
			<parameter id="A_neuq_mt_chubut.pInv" value="0.5" lower="0.0" upper="1.0"/>
		</proportionInvariant>
	</siteModel>

	<!-- The general time reversible (GTR) substitution model                    -->
	<gtrModel id="A_neuq_nu_ANT_Chubut.gtr">
		<frequencies>
			<frequencyModel dataType="nucleotide">
				<frequencies>
					<parameter id="A_neuq_nu_ANT_Chubut.frequencies" value="0.25 0.25 0.25 0.25"
				</frequencies>
			</frequencyModel>
		</frequencies>
		<rateAC>
			<parameter id="A_neuq_nu_ANT_Chubut.ac" value="1.0" lower="0.0" upper="Infinity"/>
		</rateAC>
		<rateAG>
			<parameter id="A_neuq_nu_ANT_Chubut.ag" value="1.0" lower="0.0" upper="Infinity"/>
		</rateAG>
		<rateAT>
			<parameter id="A_neuq_nu_ANT_Chubut.at" value="1.0" lower="0.0" upper="Infinity"/>
		</rateAT>
		<rateCG>
			<parameter id="A_neuq_nu_ANT_Chubut.cg" value="1.0" lower="0.0" upper="Infinity"/>
		</rateCG>
		<rateGT>
			<parameter id="A_neuq_nu_ANT_Chubut.gt" value="1.0" lower="0.0" upper="Infinity"/>
		</rateGT>
```

```
    </gtrModel>

    <!-- site model                                                              -->
    <siteModel id="A_neuq_nu_ANT_Chubut.siteModel">
        <substitutionModel>
            <gtrModel idref="A_neuq_nu_ANT_Chubut.gtr"/>
        </substitutionModel>
        <proportionInvariant>
            <parameter id="A_neuq_nu_ANT_Chubut.pInv" value="0.5" lower="0.0" upper="1.0"/>
        </proportionInvariant>
    </siteModel>

    <!-- The general time reversible (GTR) substitution model                    -->
    <gtrModel id="A_neuq_nu_EF1Exon_Chubut.gtr">
        <frequencies>
            <frequencyModel dataType="nucleotide">
                <frequencies>
                    <parameter id="A_neuq_nu_EF1Exon_Chubut.frequencies" value="0.25 0.25 0.25 0
                </frequencies>
            </frequencyModel>
        </frequencies>
        <rateAC>
            <parameter id="A_neuq_nu_EF1Exon_Chubut.ac" value="1.0" lower="0.0" upper="Infinity"
        </rateAC>
        <rateAG>
            <parameter id="A_neuq_nu_EF1Exon_Chubut.ag" value="1.0" lower="0.0" upper="Infinity"
        </rateAG>
        <rateAT>
            <parameter id="A_neuq_nu_EF1Exon_Chubut.at" value="1.0" lower="0.0" upper="Infinity"
        </rateAT>
        <rateCG>
            <parameter id="A_neuq_nu_EF1Exon_Chubut.cg" value="1.0" lower="0.0" upper="Infinity"
        </rateCG>
        <rateGT>
            <parameter id="A_neuq_nu_EF1Exon_Chubut.gt" value="1.0" lower="0.0" upper="Infinity"
        </rateGT>
    </gtrModel>

    <!-- site model                                                              -->
    <siteModel id="A_neuq_nu_EF1Exon_Chubut.siteModel">
        <substitutionModel>
            <gtrModel idref="A_neuq_nu_EF1Exon_Chubut.gtr"/>
        </substitutionModel>
    </siteModel>

    <!-- The general time reversible (GTR) substitution model                    -->
    <gtrModel id="A_neuq_nu_EF1Intron_Chubut.gtr">
        <frequencies>
            <frequencyModel dataType="nucleotide">
                <frequencies>
                    <parameter id="A_neuq_nu_EF1Intron_Chubut.frequencies" value="0.25 0.25 0.25
                </frequencies>
            </frequencyModel>
        </frequencies>
        <rateAC>
            <parameter id="A_neuq_nu_EF1Intron_Chubut.ac" value="1.0" lower="0.0" upper="Infinit
        </rateAC>
```

```
        <rateAG>
            <parameter id="A_neuq_nu_EF1Intron_Chubut.ag" value="1.0" lower="0.0" upper="Infinit
        </rateAG>
        <rateAT>
            <parameter id="A_neuq_nu_EF1Intron_Chubut.at" value="1.0" lower="0.0" upper="Infinit
        </rateAT>
        <rateCG>
            <parameter id="A_neuq_nu_EF1Intron_Chubut.cg" value="1.0" lower="0.0" upper="Infinit
        </rateCG>
        <rateGT>
            <parameter id="A_neuq_nu_EF1Intron_Chubut.gt" value="1.0" lower="0.0" upper="Infinit
        </rateGT>
    </gtrModel>

    <!-- site model                                                                    -->
    <siteModel id="A_neuq_nu_EF1Intron_Chubut.siteModel">
        <substitutionModel>
            <gtrModel idref="A_neuq_nu_EF1Intron_Chubut.gtr"/>
        </substitutionModel>
    </siteModel>
    <treeLikelihood id="A_neuq_mt_chubut.treeLikelihood" useAmbiguities="false">
        <patterns idref="A_neuq_mt_chubut.patterns"/>
        <treeModel idref="A_neuq_mt_chubut.treeModel"/>
        <siteModel idref="A_neuq_mt_chubut.siteModel"/>
        <discretizedBranchRates idref="A_neuq_mt_chubut.branchRates"/>
    </treeLikelihood>
    <treeLikelihood id="A_neuq_nu_ANT_Chubut.treeLikelihood" useAmbiguities="false">
        <patterns idref="A_neuq_nu_ANT_Chubut.patterns"/>
        <treeModel idref="A_neuq_nu_ANT_Chubut.treeModel"/>
        <siteModel idref="A_neuq_nu_ANT_Chubut.siteModel"/>
        <discretizedBranchRates idref="A_neuq_nu_ANT_Chubut.branchRates"/>
    </treeLikelihood>
    <treeLikelihood id="A_neuq_nu_EF1Exon_Chubut.treeLikelihood" useAmbiguities="false">
        <patterns idref="A_neuq_nu_EF1Exon_Chubut.patterns"/>
        <treeModel idref="A_neuq_nu_EF1Exon_Chubut.treeModel"/>
        <siteModel idref="A_neuq_nu_EF1Exon_Chubut.siteModel"/>
        <discretizedBranchRates idref="A_neuq_nu_EF1Exon_Chubut.branchRates"/>
    </treeLikelihood>
    <treeLikelihood id="A_neuq_nu_EF1Intron_Chubut.treeLikelihood" useAmbiguities="false">
        <patterns idref="A_neuq_nu_EF1Intron_Chubut.patterns"/>
        <treeModel idref="A_neuq_nu_EF1Intron_Chubut.treeModel"/>
        <siteModel idref="A_neuq_nu_EF1Intron_Chubut.siteModel"/>
        <discretizedBranchRates idref="A_neuq_nu_EF1Intron_Chubut.branchRates"/>
    </treeLikelihood>

    <!-- Define operators                                                              -->
    <operators id="operators">
        <scaleOperator scaleFactor="0.75" weight="0.1">
            <parameter idref="A_neuq_mt_chubut.kappa"/>
        </scaleOperator>
        <deltaExchange delta="0.01" weight="0.1">
            <parameter idref="A_neuq_mt_chubut.frequencies"/>
        </deltaExchange>
        <scaleOperator scaleFactor="0.75" weight="0.1">
            <parameter idref="A_neuq_mt_chubut.alpha"/>
        </scaleOperator>
        <scaleOperator scaleFactor="0.75" weight="0.1">
```

```
            <parameter idref="A_neuq_mt_chubut.pInv"/>
        </scaleOperator>
        <scaleOperator scaleFactor="0.75" weight="0.1">
            <parameter idref="A_neuq_nu_ANT_Chubut.ac"/>
        </scaleOperator>
        <scaleOperator scaleFactor="0.75" weight="0.1">
            <parameter idref="A_neuq_nu_ANT_Chubut.ag"/>
        </scaleOperator>
        <scaleOperator scaleFactor="0.75" weight="0.1">
            <parameter idref="A_neuq_nu_ANT_Chubut.at"/>
        </scaleOperator>
        <scaleOperator scaleFactor="0.75" weight="0.1">
            <parameter idref="A_neuq_nu_ANT_Chubut.cg"/>
        </scaleOperator>
        <scaleOperator scaleFactor="0.75" weight="0.1">
            <parameter idref="A_neuq_nu_ANT_Chubut.gt"/>
        </scaleOperator>
        <deltaExchange delta="0.01" weight="0.1">
            <parameter idref="A_neuq_nu_ANT_Chubut.frequencies"/>
        </deltaExchange>
        <scaleOperator scaleFactor="0.75" weight="0.1">
            <parameter idref="A_neuq_nu_ANT_Chubut.pInv"/>
        </scaleOperator>
        <deltaExchange delta="0.01" weight="0.1">
            <parameter idref="A_neuq_nu_EF1Intron_Chubut.frequencies"/>
        </deltaExchange>
        <scaleOperator scaleFactor="0.75" weight="3">
            <parameter idref="A_neuq_mt_chubut.ucld.stdev"/>
        </scaleOperator>
        <scaleOperator scaleFactor="0.75" weight="3">
            <parameter idref="A_neuq_nu_ANT_Chubut.ucld.mean"/>
        </scaleOperator>
        <scaleOperator scaleFactor="0.75" weight="3">
            <parameter idref="A_neuq_nu_ANT_Chubut.ucld.stdev"/>
        </scaleOperator>
        <scaleOperator scaleFactor="0.75" weight="3">
            <parameter idref="A_neuq_nu_EF1Exon_Chubut.ucld.mean"/>
        </scaleOperator>
        <scaleOperator scaleFactor="0.75" weight="3">
            <parameter idref="A_neuq_nu_EF1Exon_Chubut.ucld.stdev"/>
        </scaleOperator>
        <scaleOperator scaleFactor="0.75" weight="3">
            <parameter idref="A_neuq_nu_EF1Intron_Chubut.ucld.mean"/>
        </scaleOperator>
        <scaleOperator scaleFactor="0.75" weight="3">
            <parameter idref="A_neuq_nu_EF1Intron_Chubut.ucld.stdev"/>
        </scaleOperator>
        <upDownOperator scaleFactor="0.75" weight="30">
            <up>
                <parameter idref="A_neuq_nu_ANT_Chubut.ucld.mean"/>
                <parameter idref="A_neuq_nu_EF1Exon_Chubut.ucld.mean"/>
                <parameter idref="A_neuq_nu_EF1Intron_Chubut.ucld.mean"/>
            </up>
            <down>
                <parameter idref="demographic.popSize"/>
                <parameter idref="A_neuq_mt_chubut.treeModel.allInternalNodeHeights"/>
                <parameter idref="A_neuq_nu_ANT_Chubut.treeModel.allInternalNodeHeights"/>
```

```
                <parameter idref="A_neuq_nu_EF1Exon_Chubut.treeModel.allInternalNodeHeights"/>
                <parameter idref="A_neuq_nu_EF1Intron_Chubut.treeModel.allInternalNodeHeights"/>
            </down>
        </upDownOperator>
        <subtreeSlide size="0.0017000000000000001" gaussian="true" weight="15">
            <treeModel idref="A_neuq_mt_chubut.treeModel"/>
        </subtreeSlide>
        <narrowExchange weight="15">
            <treeModel idref="A_neuq_mt_chubut.treeModel"/>
        </narrowExchange>
        <wideExchange weight="3">
            <treeModel idref="A_neuq_mt_chubut.treeModel"/>
        </wideExchange>
        <wilsonBalding weight="3">
            <treeModel idref="A_neuq_mt_chubut.treeModel"/>
        </wilsonBalding>
        <scaleOperator scaleFactor="0.75" weight="3">
            <parameter idref="A_neuq_mt_chubut.treeModel.rootHeight"/>
        </scaleOperator>
        <uniformOperator weight="30">
            <parameter idref="A_neuq_mt_chubut.treeModel.internalNodeHeights"/>
        </uniformOperator>
        <subtreeSlide size="1.1999999999999999E-4" gaussian="true" weight="15">
            <treeModel idref="A_neuq_nu_ANT_Chubut.treeModel"/>
        </subtreeSlide>
        <narrowExchange weight="15">
            <treeModel idref="A_neuq_nu_ANT_Chubut.treeModel"/>
        </narrowExchange>
        <wideExchange weight="3">
            <treeModel idref="A_neuq_nu_ANT_Chubut.treeModel"/>
        </wideExchange>
        <wilsonBalding weight="3">
            <treeModel idref="A_neuq_nu_ANT_Chubut.treeModel"/>
        </wilsonBalding>
        <scaleOperator scaleFactor="0.75" weight="3">
            <parameter idref="A_neuq_nu_ANT_Chubut.treeModel.rootHeight"/>
        </scaleOperator>
        <uniformOperator weight="30">
            <parameter idref="A_neuq_nu_ANT_Chubut.treeModel.internalNodeHeights"/>
        </uniformOperator>
        <subtreeSlide size="0.0001" gaussian="true" weight="15">
            <treeModel idref="A_neuq_nu_EF1Exon_Chubut.treeModel"/>
        </subtreeSlide>
        <narrowExchange weight="15">
            <treeModel idref="A_neuq_nu_EF1Exon_Chubut.treeModel"/>
        </narrowExchange>
        <wideExchange weight="3">
            <treeModel idref="A_neuq_nu_EF1Exon_Chubut.treeModel"/>
        </wideExchange>
        <wilsonBalding weight="3">
            <treeModel idref="A_neuq_nu_EF1Exon_Chubut.treeModel"/>
        </wilsonBalding>
        <scaleOperator scaleFactor="0.75" weight="3">
            <parameter idref="A_neuq_nu_EF1Exon_Chubut.treeModel.rootHeight"/>
        </scaleOperator>
        <uniformOperator weight="30">
            <parameter idref="A_neuq_nu_EF1Exon_Chubut.treeModel.internalNodeHeights"/>
```

```
        </uniformOperator>
        <subtreeSlide size="0.001" gaussian="true" weight="15">
            <treeModel idref="A_neuq_nu_EF1Intron_Chubut.treeModel"/>
        </subtreeSlide>
        <narrowExchange weight="15">
            <treeModel idref="A_neuq_nu_EF1Intron_Chubut.treeModel"/>
        </narrowExchange>
        <wideExchange weight="3">
            <treeModel idref="A_neuq_nu_EF1Intron_Chubut.treeModel"/>
        </wideExchange>
        <wilsonBalding weight="3">
            <treeModel idref="A_neuq_nu_EF1Intron_Chubut.treeModel"/>
        </wilsonBalding>
        <scaleOperator scaleFactor="0.75" weight="3">
            <parameter idref="A_neuq_nu_EF1Intron_Chubut.treeModel.rootHeight"/>
        </scaleOperator>
        <uniformOperator weight="30">
            <parameter idref="A_neuq_nu_EF1Intron_Chubut.treeModel.internalNodeHeights"/>
        </uniformOperator>
        <scaleOperator scaleFactor="0.9" weight="3">
            <parameter idref="demographic.populationMean"/>
        </scaleOperator>
        <sampleNonActiveOperator weight="15">
            <distribution>
                <parameter idref="demographic.populationMeanDist"/>
            </distribution>
            <data>
                <parameter idref="demographic.popSize"/>
            </data>
            <indicators>
                <parameter idref="demographic.indicators"/>
            </indicators>
        </sampleNonActiveOperator>
        <bitFlipOperator weight="30">
            <parameter idref="demographic.indicators"/>
        </bitFlipOperator>
        <scaleOperator scaleFactor="0.5" weight="6">
            <parameter idref="demographic.popSize"/>
            <indicators pickoneprob="1.0">
                <parameter idref="demographic.indicators"/>
            </indicators>
        </scaleOperator>
        <upDownOperator scaleFactor="0.75" weight="3">
            <up>
            </up>
            <down>
                <parameter idref="A_neuq_mt_chubut.treeModel.allInternalNodeHeights"/>
            </down>
        </upDownOperator>
        <swapOperator size="1" weight="10" autoOptimize="false">
            <parameter idref="A_neuq_mt_chubut.branchRates.categories"/>
        </swapOperator>
        <randomWalkIntegerOperator windowSize="1" weight="10">
            <parameter idref="A_neuq_mt_chubut.branchRates.categories"/>
        </randomWalkIntegerOperator>
        <uniformIntegerOperator weight="10">
            <parameter idref="A_neuq_mt_chubut.branchRates.categories"/>
```

```xml
        </uniformIntegerOperator>
        <upDownOperator scaleFactor="0.75" weight="3">
            <up>
                <parameter idref="A_neuq_nu_ANT_Chubut.ucld.mean"/>
            </up>
            <down>
                <parameter idref="A_neuq_nu_ANT_Chubut.treeModel.allInternalNodeHeights"/>
            </down>
        </upDownOperator>
        <swapOperator size="1" weight="10" autoOptimize="false">
            <parameter idref="A_neuq_nu_ANT_Chubut.branchRates.categories"/>
        </swapOperator>
        <randomWalkIntegerOperator windowSize="1" weight="10">
            <parameter idref="A_neuq_nu_ANT_Chubut.branchRates.categories"/>
        </randomWalkIntegerOperator>
        <uniformIntegerOperator weight="10">
            <parameter idref="A_neuq_nu_ANT_Chubut.branchRates.categories"/>
        </uniformIntegerOperator>
        <upDownOperator scaleFactor="0.75" weight="3">
            <up>
                <parameter idref="A_neuq_nu_EF1Exon_Chubut.ucld.mean"/>
            </up>
            <down>
                <parameter idref="A_neuq_nu_EF1Exon_Chubut.treeModel.allInternalNodeHeights"/>
            </down>
        </upDownOperator>
        <swapOperator size="1" weight="10" autoOptimize="false">
            <parameter idref="A_neuq_nu_EF1Exon_Chubut.branchRates.categories"/>
        </swapOperator>
        <randomWalkIntegerOperator windowSize="1" weight="10">
            <parameter idref="A_neuq_nu_EF1Exon_Chubut.branchRates.categories"/>
        </randomWalkIntegerOperator>
        <uniformIntegerOperator weight="10">
            <parameter idref="A_neuq_nu_EF1Exon_Chubut.branchRates.categories"/>
        </uniformIntegerOperator>
        <upDownOperator scaleFactor="0.75" weight="3">
            <up>
                <parameter idref="A_neuq_nu_EF1Intron_Chubut.ucld.mean"/>
            </up>
            <down>
                <parameter idref="A_neuq_nu_EF1Intron_Chubut.treeModel.allInternalNodeHeights"/>
            </down>
        </upDownOperator>
        <swapOperator size="1" weight="10" autoOptimize="false">
            <parameter idref="A_neuq_nu_EF1Intron_Chubut.branchRates.categories"/>
        </swapOperator>
        <randomWalkIntegerOperator windowSize="1" weight="10">
            <parameter idref="A_neuq_nu_EF1Intron_Chubut.branchRates.categories"/>
        </randomWalkIntegerOperator>
        <uniformIntegerOperator weight="10">
            <parameter idref="A_neuq_nu_EF1Intron_Chubut.branchRates.categories"/>
        </uniformIntegerOperator>
    </operators>

    <!-- Define MCMC                                                             -->
    <mcmc id="mcmc" chainLength="50000000" autoOptimize="true">
        <posterior id="posterior">
```

```
            <prior id="prior">
                <logNormalPrior mean="1.0" stdev="1.25" offset="0.0" meanInRealSpace="false">
                    <parameter idref="A_neuq_mt_chubut.kappa"/>
                </logNormalPrior>
                <gammaPrior shape="0.05" scale="10.0" offset="0.0">
                    <parameter idref="A_neuq_nu_ANT_Chubut.ac"/>
                </gammaPrior>
                <gammaPrior shape="0.05" scale="20.0" offset="0.0">
                    <parameter idref="A_neuq_nu_ANT_Chubut.ag"/>
                </gammaPrior>
                <gammaPrior shape="0.05" scale="10.0" offset="0.0">
                    <parameter idref="A_neuq_nu_ANT_Chubut.at"/>
                </gammaPrior>
                <gammaPrior shape="0.05" scale="10.0" offset="0.0">
                    <parameter idref="A_neuq_nu_ANT_Chubut.cg"/>
                </gammaPrior>
                <gammaPrior shape="0.05" scale="10.0" offset="0.0">
                    <parameter idref="A_neuq_nu_ANT_Chubut.gt"/>
                </gammaPrior>
                <gammaPrior shape="0.05" scale="10.0" offset="0.0">
                    <parameter idref="A_neuq_nu_EF1Exon_Chubut.ac"/>
                </gammaPrior>
                <gammaPrior shape="0.05" scale="20.0" offset="0.0">
                    <parameter idref="A_neuq_nu_EF1Exon_Chubut.ag"/>
                </gammaPrior>
                <gammaPrior shape="0.05" scale="10.0" offset="0.0">
                    <parameter idref="A_neuq_nu_EF1Exon_Chubut.at"/>
                </gammaPrior>
                <gammaPrior shape="0.05" scale="10.0" offset="0.0">
                    <parameter idref="A_neuq_nu_EF1Exon_Chubut.cg"/>
                </gammaPrior>
                <gammaPrior shape="0.05" scale="10.0" offset="0.0">
                    <parameter idref="A_neuq_nu_EF1Exon_Chubut.gt"/>
                </gammaPrior>
                <gammaPrior shape="0.05" scale="10.0" offset="0.0">
                    <parameter idref="A_neuq_nu_EF1Intron_Chubut.ac"/>
                </gammaPrior>
                <gammaPrior shape="0.05" scale="20.0" offset="0.0">
                    <parameter idref="A_neuq_nu_EF1Intron_Chubut.ag"/>
                </gammaPrior>
                <gammaPrior shape="0.05" scale="10.0" offset="0.0">
                    <parameter idref="A_neuq_nu_EF1Intron_Chubut.at"/>
                </gammaPrior>
                <gammaPrior shape="0.05" scale="10.0" offset="0.0">
                    <parameter idref="A_neuq_nu_EF1Intron_Chubut.cg"/>
                </gammaPrior>
                <gammaPrior shape="0.05" scale="10.0" offset="0.0">
                    <parameter idref="A_neuq_nu_EF1Intron_Chubut.gt"/>
                </gammaPrior>
                <exponentialPrior mean="0.3333333333333333" offset="0.0">
                    <parameter idref="A_neuq_mt_chubut.ucld.stdev"/>
                </exponentialPrior>
                <exponentialPrior mean="0.3333333333333333" offset="0.0">
                    <parameter idref="A_neuq_nu_ANT_Chubut.ucld.stdev"/>
                </exponentialPrior>
                <logNormalPrior mean="0.0" stdev="1.0" offset="0.0" meanInRealSpace="false">
                    <parameter idref="A_neuq_nu_ANT_Chubut.ucld.mean"/>
```

```
                </logNormalPrior>
                <exponentialPrior mean="0.3333333333333333" offset="0.0">
                    <parameter idref="A_neuq_nu_EF1Exon_Chubut.ucld.stdev"/>
                </exponentialPrior>
                <logNormalPrior mean="0.0" stdev="1.0" offset="0.0" meanInRealSpace="false">
                    <parameter idref="A_neuq_nu_EF1Exon_Chubut.ucld.mean"/>
                </logNormalPrior>
                <exponentialPrior mean="0.3333333333333333" offset="0.0">
                    <parameter idref="A_neuq_nu_EF1Intron_Chubut.ucld.stdev"/>
                </exponentialPrior>
                <logNormalPrior mean="0.0" stdev="1.0" offset="0.0" meanInRealSpace="false">
                    <parameter idref="A_neuq_nu_EF1Intron_Chubut.ucld.mean"/>
                </logNormalPrior>
                <poissonPrior mean="0.6931471805599453" offset="0.0">
                    <statistic idref="demographic.populationSizeChanges"/>
                </poissonPrior>
                <oneOnXPrior>
                    <parameter idref="demographic.populationMean"/>
                </oneOnXPrior>
                <coalescentLikelihood idref="coalescent"/>
                <mixedDistributionLikelihood>
                    <distribution0>
                        <exponentialDistributionModel idref="demographic.populationMeanDist"/>
                    </distribution0>
                    <distribution1>
                        <exponentialDistributionModel idref="demographic.populationMeanDist"/>
                    </distribution1>
                    <data>
                        <parameter idref="demographic.popSize"/>
                    </data>
                    <indicators>
                        <parameter idref="demographic.indicators"/>
                    </indicators>
                </mixedDistributionLikelihood>
            </prior>
            <likelihood id="likelihood">
                <treeLikelihood idref="A_neuq_mt_chubut.treeLikelihood"/>
                <treeLikelihood idref="A_neuq_nu_ANT_Chubut.treeLikelihood"/>
                <treeLikelihood idref="A_neuq_nu_EF1Exon_Chubut.treeLikelihood"/>
                <treeLikelihood idref="A_neuq_nu_EF1Intron_Chubut.treeLikelihood"/>
            </likelihood>
        </posterior>
        <operators idref="operators"/>

        <!-- write log to screen                                                  -->
        <log id="screenLog" logEvery="5000">
            <column label="Posterior" dp="4" width="12">
                <posterior idref="posterior"/>
            </column>
            <column label="Prior" dp="4" width="12">
                <prior idref="prior"/>
            </column>
            <column label="Likelihood" dp="4" width="12">
                <likelihood idref="likelihood"/>
            </column>
            <column label="A_neuq_mt_chubut.rootHeight" sf="6" width="12">
                <parameter idref="A_neuq_mt_chubut.treeModel.rootHeight"/>
```

```
        </column>
        <column label="A_neuq_nu_ANT_Chubut.rootHeight" sf="6" width="12">
            <parameter idref="A_neuq_nu_ANT_Chubut.treeModel.rootHeight"/>
        </column>
        <column label="A_neuq_nu_EF1Exon_Chubut.rootHeight" sf="6" width="12">
            <parameter idref="A_neuq_nu_EF1Exon_Chubut.treeModel.rootHeight"/>
        </column>
        <column label="A_neuq_nu_EF1Intron_Chubut.rootHeight" sf="6" width="12">
            <parameter idref="A_neuq_nu_EF1Intron_Chubut.treeModel.rootHeight"/>
        </column>
        <column label="A_neuq_mt_chubut.ucld.mean" sf="6" width="12">
            <parameter idref="A_neuq_mt_chubut.ucld.mean"/>
        </column>
        <column label="A_neuq_nu_ANT_Chubut.ucld.mean" sf="6" width="12">
            <parameter idref="A_neuq_nu_ANT_Chubut.ucld.mean"/>
        </column>
        <column label="A_neuq_nu_EF1Exon_Chubut.ucld.mean" sf="6" width="12">
            <parameter idref="A_neuq_nu_EF1Exon_Chubut.ucld.mean"/>
        </column>
        <column label="A_neuq_nu_EF1Intron_Chubut.ucld.mean" sf="6" width="12">
            <parameter idref="A_neuq_nu_EF1Intron_Chubut.ucld.mean"/>
        </column>
    </log>

    <!-- write log to file                                                    -->
    <log id="fileLog" logEvery="2000" fileName="A_neuq_ALL_chubut.log" overwrite="false">
        <posterior idref="posterior"/>
        <prior idref="prior"/>
        <likelihood idref="likelihood"/>
        <parameter idref="A_neuq_mt_chubut.treeModel.rootHeight"/>
        <parameter idref="A_neuq_nu_ANT_Chubut.treeModel.rootHeight"/>
        <parameter idref="A_neuq_nu_EF1Exon_Chubut.treeModel.rootHeight"/>
        <parameter idref="A_neuq_nu_EF1Intron_Chubut.treeModel.rootHeight"/>
        <sumStatistic idref="demographic.populationSizeChanges"/>
        <parameter idref="demographic.populationMean"/>
        <parameter idref="demographic.popSize"/>
        <parameter idref="demographic.indicators"/>
        <parameter idref="A_neuq_mt_chubut.kappa"/>
        <parameter idref="A_neuq_mt_chubut.frequencies"/>
        <parameter idref="A_neuq_mt_chubut.alpha"/>
        <parameter idref="A_neuq_mt_chubut.pInv"/>
        <parameter idref="A_neuq_nu_ANT_Chubut.ac"/>
        <parameter idref="A_neuq_nu_ANT_Chubut.ag"/>
        <parameter idref="A_neuq_nu_ANT_Chubut.at"/>
        <parameter idref="A_neuq_nu_ANT_Chubut.cg"/>
        <parameter idref="A_neuq_nu_ANT_Chubut.gt"/>
        <parameter idref="A_neuq_nu_ANT_Chubut.frequencies"/>
        <parameter idref="A_neuq_nu_ANT_Chubut.pInv"/>
        <parameter idref="A_neuq_nu_EF1Exon_Chubut.ac"/>
        <parameter idref="A_neuq_nu_EF1Exon_Chubut.ag"/>
        <parameter idref="A_neuq_nu_EF1Exon_Chubut.at"/>
        <parameter idref="A_neuq_nu_EF1Exon_Chubut.cg"/>
        <parameter idref="A_neuq_nu_EF1Exon_Chubut.gt"/>
        <parameter idref="A_neuq_nu_EF1Intron_Chubut.ac"/>
        <parameter idref="A_neuq_nu_EF1Intron_Chubut.ag"/>
        <parameter idref="A_neuq_nu_EF1Intron_Chubut.at"/>
        <parameter idref="A_neuq_nu_EF1Intron_Chubut.cg"/>
```

```
            <parameter idref="A_neuq_nu_EF1Intron_Chubut.gt"/>
            <parameter idref="A_neuq_nu_EF1Intron_Chubut.frequencies"/>
            <parameter idref="A_neuq_mt_chubut.ucld.mean"/>
            <parameter idref="A_neuq_mt_chubut.ucld.stdev"/>
            <parameter idref="A_neuq_nu_ANT_Chubut.ucld.mean"/>
            <parameter idref="A_neuq_nu_ANT_Chubut.ucld.stdev"/>
            <parameter idref="A_neuq_nu_EF1Exon_Chubut.ucld.mean"/>
            <parameter idref="A_neuq_nu_EF1Exon_Chubut.ucld.stdev"/>
            <parameter idref="A_neuq_nu_EF1Intron_Chubut.ucld.mean"/>
            <parameter idref="A_neuq_nu_EF1Intron_Chubut.ucld.stdev"/>
            <rateStatistic idref="A_neuq_mt_chubut.meanRate"/>
            <rateStatistic idref="A_neuq_mt_chubut.coefficientOfVariation"/>
            <rateCovarianceStatistic idref="A_neuq_mt_chubut.covariance"/>
            <rateStatistic idref="A_neuq_nu_ANT_Chubut.meanRate"/>
            <rateStatistic idref="A_neuq_nu_ANT_Chubut.coefficientOfVariation"/>
            <rateCovarianceStatistic idref="A_neuq_nu_ANT_Chubut.covariance"/>
            <rateStatistic idref="A_neuq_nu_EF1Exon_Chubut.meanRate"/>
            <rateStatistic idref="A_neuq_nu_EF1Exon_Chubut.coefficientOfVariation"/>
            <rateCovarianceStatistic idref="A_neuq_nu_EF1Exon_Chubut.covariance"/>
            <rateStatistic idref="A_neuq_nu_EF1Intron_Chubut.meanRate"/>
            <rateStatistic idref="A_neuq_nu_EF1Intron_Chubut.coefficientOfVariation"/>
            <rateCovarianceStatistic idref="A_neuq_nu_EF1Intron_Chubut.covariance"/>
            <treeLikelihood idref="A_neuq_mt_chubut.treeLikelihood"/>
            <treeLikelihood idref="A_neuq_nu_ANT_Chubut.treeLikelihood"/>
            <treeLikelihood idref="A_neuq_nu_EF1Exon_Chubut.treeLikelihood"/>
            <treeLikelihood idref="A_neuq_nu_EF1Intron_Chubut.treeLikelihood"/>
            <coalescentLikelihood idref="coalescent"/>
        </log>

        <!-- write tree log to file                                      -->
        <logTree id="A_neuq_mt_chubut.treeFileLog" logEvery="1000" nexusFormat="true" fileName="
            <treeModel idref="A_neuq_mt_chubut.treeModel"/>
            <discretizedBranchRates idref="A_neuq_mt_chubut.branchRates"/>
            <posterior idref="posterior"/>
        </logTree>
        <logTree id="A_neuq_nu_ANT_Chubut.treeFileLog" logEvery="1000" nexusFormat="true" fileNa
            <treeModel idref="A_neuq_nu_ANT_Chubut.treeModel"/>
            <discretizedBranchRates idref="A_neuq_nu_ANT_Chubut.branchRates"/>
            <posterior idref="posterior"/>
        </logTree>
        <logTree id="A_neuq_nu_EF1Exon_Chubut.treeFileLog" logEvery="1000" nexusFormat="true" fi
            <treeModel idref="A_neuq_nu_EF1Exon_Chubut.treeModel"/>
            <discretizedBranchRates idref="A_neuq_nu_EF1Exon_Chubut.branchRates"/>
            <posterior idref="posterior"/>
        </logTree>
        <logTree id="A_neuq_nu_EF1Intron_Chubut.treeFileLog" logEvery="1000" nexusFormat="true"
            <treeModel idref="A_neuq_nu_EF1Intron_Chubut.treeModel"/>
            <discretizedBranchRates idref="A_neuq_nu_EF1Intron_Chubut.branchRates"/>
            <posterior idref="posterior"/>
        </logTree>
    </mcmc>
    <report>
        <property name="timer">
            <mcmc idref="mcmc"/>
        </property>
    </report>
    <VDAnalysis id="demographic.analysis" burnIn="0.25" useMidpoints="true">
```

```
        <logFileName>
            A_neuq_ALL_chubut.log
        </logFileName>
        <treeFileNames>
            <treeOfLoci>
                A_neuq_ALL_chubut.A_neuq_mt_chubut.trees
            </treeOfLoci>
            <treeOfLoci>
                A_neuq_ALL_chubut.A_neuq_nu_ANT_Chubut.trees
            </treeOfLoci>
            <treeOfLoci>
                A_neuq_ALL_chubut.A_neuq_nu_EF1Exon_Chubut.trees
            </treeOfLoci>
            <treeOfLoci>
                A_neuq_ALL_chubut.A_neuq_nu_EF1Intron_Chubut.trees
            </treeOfLoci>
        </treeFileNames>
        <populationModelType>
            linear
        </populationModelType>
        <populationFirstColumn>
            demographic.popSize1
        </populationFirstColumn>
        <indicatorsFirstColumn>
            demographic.indicators1
        </indicatorsFirstColumn>
    </VDAnalysis>
    <CSVexport fileName="A_neuq_ALL_chubut.csv" separator=",">
        <columns>
            <VDAnalysis idref="demographic.analysis"/>
        </columns>
    </CSVexport>
</beast>
```
